# Deep Active Inference and Scene Construction

**DOI:** 10.3389/frai.2020.509354

**Published:** 2020-10-28

**Authors:** R. Conor Heins, M. Berk Mirza, Thomas Parr, Karl Friston, Igor Kagan, Arezoo Pooresmaeili

**Affiliations:** ^1^Department of Collective Behaviour, Max Planck Institute for Animal Behavior, Konstanz, Germany; ^2^Perception and Cognition Group, European Neuroscience Institute, A Joint Initiative of the University Medical Centre Göttingen and the Max-Planck-Society, Göttingen, Germany; ^3^Leibniz Science Campus “Primate Cognition”, Göttingen, Germany; ^4^Department of Neuroimaging, Institute of Psychiatry, Psychology and Neuroscience, King's College London, London, United Kingdom; ^5^The National Institute for Health Research (NIHR) Maudsley Biomedical Research Centre (BRC) at South London and Maudsley National Health Service (NHS) Foundation Trust and The Institute of Psychiatry, Psychology and Neuroscience, King's College London, London, United Kingdom; ^6^Wellcome Centre for Human Neuroimaging, University College London, London, United Kingdom; ^7^Decision and Awareness Group, Cognitive Neuroscience Laboratory, German Primate Centre (DPZ), Göttingen, Germany

**Keywords:** active inference, visual foraging, Bayesian brain, hierarchical inference, free energy, epistemic value, random dot motion

## Abstract

Adaptive agents must act in intrinsically uncertain environments with complex latent structure. Here, we elaborate a model of visual foraging—in a hierarchical context—wherein agents infer a higher-order visual pattern (a “scene”) by sequentially sampling ambiguous cues. Inspired by previous models of scene construction—that cast perception and action as consequences of approximate Bayesian inference—we use active inference to simulate decisions of agents categorizing a scene in a hierarchically-structured setting. Under active inference, agents develop probabilistic beliefs about their environment, while actively sampling it to maximize the evidence for their internal generative model. This approximate evidence maximization (i.e., self-evidencing) comprises drives to both maximize rewards and resolve uncertainty about hidden states. This is realized via minimization of a free energy functional of posterior beliefs about both the world as well as the actions used to sample or perturb it, corresponding to perception and action, respectively. We show that active inference, in the context of hierarchical scene construction, gives rise to many empirical evidence accumulation phenomena, such as noise-sensitive reaction times and epistemic saccades. We explain these behaviors in terms of the principled drives that constitute the *expected free energy*, the key quantity for evaluating policies under active inference. In addition, we report novel behaviors exhibited by these active inference agents that furnish new predictions for research on evidence accumulation and perceptual decision-making. We discuss the implications of this hierarchical active inference scheme for tasks that require planned sequences of information-gathering actions to infer compositional latent structure (such as visual scene construction and sentence comprehension). This work sets the stage for future experiments to investigate active inference in relation to other formulations of evidence accumulation (e.g., drift-diffusion models) in tasks that require planning in uncertain environments with higher-order structure.

## 1. Introduction

Our daily life is full of complex sensory scenarios that can be described as examples of “scene construction” (Hassabis and Maguire, 2007; Zeidman et al., 2015; Mirza et al., 2016). In its most abstract sense, scene construction describes the act of inferring a latent variable (or “scene”) given a set of (potentially ambiguous) sensory cues. Sentence comprehension is a prime example of scene construction: individual words are inspected in isolation, but after reading a sequence one is able to abduce the overall meaning of the sentence that the words are embedded within (Tanenhaus et al., 1995; Narayanan and Jurafsky, 1998; Ferro et al., 2010). This can be cast as a form of hierarchical inference in which low-level evidence (e.g., words) is actively accumulated over time to support disambiguation of high-level hypotheses (e.g., possible sentence meanings).

We investigate hierarchical belief-updating by modeling visual foraging as a form of scene construction, where individual images are actively sampled with saccadic eye movements in order to accumulate information and categorize the scene accurately (Yarbus, 1967; Jóhannesson et al., 2016; Mirza et al., 2016; Yang et al., 2016; Ólafsdóttir et al., 2019). In the context of scene construction, sensory uncertainty (e.g., blurry images) can limit the ability of individual cues to support inference about the overarching visual scene. Such sensory uncertainty can be partially “overridden” using prior knowledge, which might be built into the agent's internal model, innately or based on previous experience. While there is an enormous body of literature on the resolution of uncertainty with prior information (Trueswell et al., 1994; Rayner and Well, 1996; Körding and Wolpert, 2004; Stocker and Simoncelli, 2006; Girshick et al., 2011), relatively little research has examined interactions between sensory uncertainty and prior information in the context of a dynamic, active vision task like visual foraging or scene construction (with notable exceptions: e.g., Quétard et al., 2016).

Building on a previous Bayesian formulation of scene construction, in this work use we use *active inference* to model visual foraging in a hierarchical scene construction task (Friston et al., 2012a,b, 2017a; Mirza et al., 2016), and to study different types of uncertainty across distinct “layers of inference.” We present simulations of active inference agents performing hierarchical scene construction while parametrically manipulating sensory uncertainty and prior beliefs. The (sometimes counterintuitive) results of our simulations invite new perspectives on active sensing and hierarchical inference, which we discuss in the context of experimental design for both visual foraging experiments and perceptual decision-making tasks more generally. We examine the model's behavior in terms of the tension between instrumental (or utility-driven) and exploratory (epistemically-driven) drives, and how active inference explains both by appealing to a single pseudo- “value function”: the *expected free energy*.

The rest of this paper is structured as follows: first, we summarize active inference and the free energy principle, highlighting the *expected free energy*, a quantity that prescribes behavior with both goal-satisfying and information-gathering components, under the single theoretical mantle of maximizing model evidence. Next, we discuss the original model of scene construction that inspired the present work, and move on to introduce random dot motion stimuli and the ensuing ability to parametrically manipulate uncertainty across hierarchical levels, which distinguishes the current model from the original. Then we detail the Markov Decision Process generative model that our active inference agents entertain, and describe the belief-updating procedures used to invert generative models, given observed sensory data. Having appropriately set up our scene construction task, we then report the results of simulations, with differential effects of sensory uncertainty and prior belief strength appearing in several aspects of active evidence accumulation in this hierarchical environment. These computational demonstrations motivate our conclusion, where we discuss the implications of this work for experimental and theoretical studies of active sensing and evidence accumulation under uncertainty.

## 2. Free Energy Minimization and Active Inference

### 2.1. Approximate Inference via Variational Bayes

The goal of Bayesian inference is infer possible explanations for data—this means obtaining a distribution over a set of parameters *x* (the causal variables or explanations), given some observations õ, where the tilde ~ notation indicates a sequence of such observations over time $õ={\left[o_{1},o_{2},\ldots o_{T}\right]}^{T}$. Note we use the notation *x* to refer to a set of causal variables, which may include (sequences of) states $\tilde{s}$ and/or hyperparameters. This is also called calculating the posterior probability *P*(*x*|õ); it encodes the *optimal* belief about causal variables *x*, after having observed some data õ. To compute the posterior requires solving using Bayes rule:

(1) $$P(x|\tilde{o})=\frac{P(\tilde{o}|x)P(x)}{P(\tilde{o})}$$

Importantly, computing this quantity requires calculating the marginal probability *P*(õ), also known as the *evidence*:

(2) $$P(\tilde{o})=\sum_{x}P(x)P(\tilde{o}|x)$$

Solving this summation^1^ (in the continuous case, integration) quickly becomes intractable for high-dimensional models, since the evidence needs to be calculated for every possible combination of parameters *x*. The marginalization in Equation (2) renders exact Bayesian inference expensive or impossible in many cases, motivating approximate inference methods. One of the leading classes of methods for approximate inference are the variational methods (Beal, 2004; Blei et al., 2017). Variational inference circumvents the issue of exact inference by introducing an arbitrary distribution *Q*(*x*) to replace the true posterior. This replacement is often referred to as the *variational* or *approximate posterior*. By constraining the form of the variational distribution, tractable schemes exist to optimize it in a way that (approximately) maximizes evidence. This optimization occurs with respect to a quantity called the *variational free energy*, which is a computable upper-bound on *surprise*, or the negative (log) evidence −ln *P*(õ). The relationship between surprise and free energy can be shown as follows using Jensen's inequality:

(3) $$\begin{array}{l} -\text{ln }P(\tilde{o})=-\text{ln }\sum_{x}P(\tilde{o},x)\\ \;=-\text{ln }\sum_{x}Q(x)\frac{P(\tilde{o},x)}{Q(x)}\\ \;\leq -\sum_{x}Q(x)\text{ln }\frac{P(\tilde{o},x)}{Q(x)}=F\\ \Rightarrow -\text{ln }P(\tilde{o})\leq F \end{array}$$

where *F* is the variational free energy and *P*(õ, *x*) is the joint probability of observations and hidden causes, also known as the *generative model*. The free energy can itself be decomposed into:

(4) $$F={\text{D}}_{KL}[Q(x)\|P(x|\tilde{o})]-\text{ln }P(\tilde{o})$$

This decomposition allows us to see that the free energy becomes a tighter upper-bound on surprise the closer the variational distribution *Q*(*x*) comes to the true posterior *P*(*x*|õ), as measured by the Kullback-Leibler divergence^2^. When *Q*(*x*) = *P*(*x*|õ), the divergence disappears and the free energy equals the negative log evidence, rendering inference exact. Variational inference is thus often described as the conversion of an integration problem (computing the marginal likelihood of observations as in Equation (2)) into an optimization problem, wherein the parameters of the variational distribution are changed to minimize *F*:

(5) $$Q(x)=\text{arg }\underset{Q(x)}{\text{min}}F\approx P(x|\tilde{o})$$

### 2.2. Active Inference and Expected Free Energy

Having discussed the variational approximation to Bayesian inference via free energy minimization, we now turn our attention to active inference. Active inference is a framework for modeling and understanding adaptive agents, premised on the idea that agents engage in approximate Bayesian inference with respect to an internal generative model of sensory data. Crucially, under active inference both action and perception are realizations of the single drive to minimize surprise. By using variational Bayesian inference to achieve this, an active inference agent generates Bayes-optimal beliefs about sources of variation in its environment by free-energy-driven optimization of an approximate posterior *Q*(*x*). This can be analogized to the idea of perception as inference, wherein perception constitutes optimizing the parameters of an approximate posterior distribution over hidden states $Q\left(\tilde{s}|\pi \right)$, under a particular policy π^3^. In the context of neural systems, it is theorized that the parameters of these posterior beliefs about states are encoded by distributed neural activity in the agent's brain (Friston, 2008; Friston and Kiebel, 2009; Huang and Rao, 2011; Bastos et al., 2012; Parr and Friston, 2018c). Parameters of the generative model itself (such as the likelihood mapping *P*(*o*|*s*)) are hypothesized to be encoded by the network architectures, synaptic strengths, and neuromodulatory elements of the nervous system (Bogacz, 2017; Parr et al., 2018, 2019).

*Action* can also be framed as a consequence of variational Bayesian inference. Under active inference, policies (sequences of actions) correspond to sequences of “control states” —a type of hidden state that agents can directly influence. Actions are treated as samples from posterior beliefs about policies (Friston et al., 2012b). However, optimizing beliefs about policies introduces an additional complication. Optimal beliefs about hidden states $Q\left(\tilde{s}\right)$ are a function of current and past observations. However, as the instantaneous free energy is a direct function of observations, it is not immediately clear how to optimize beliefs about policies when observations from the future are not available. This motivates the introduction of the *expected free energy*, or beliefs about the free energy expected in the future when pursuing a policy π. The free energy expected at future time point τ under a policy π is given by *G*(π, τ). Replacing the expectation over hidden states and outcomes in Equation (3) with the expectation over hidden states and outcomes in the future, we have:

(6) $$G(\pi ,\tau )=\sum_{o,s}Q(o_{\tau },s_{\tau }|\pi )\text{ln }\frac{Q(s_{\tau }|\pi )}{P(o_{\tau },s_{\tau })}$$

Here, we equip the agent with the prior belief that its policies minimize the free energy expected (under their pursuit) in the future. Under Markovian assumptions on the dependence between subsequent time points in the generative model $P\left(õ,\tilde{s}|\pi \right)=\prod_{t}^{T}P\left(o_{t}|s_{t}\right)P\left(s_{t}|s_{t-1},\pi \right)$ and a mean-field factorization of the approximate posterior across time (such that $Q\left(\tilde{s}|\pi \right)=Q\left(\pi \right)\prod_{\tau =1}^{T}Q\left(s_{\tau }\right)$), we can write the prior probability of a policy as proportional to the sum of the expected free energies over time under each policy:

(7) $$P(\pi )\propto \text{exp}(-\sum_{\tau }G(\pi ,\tau ))$$

We will not derive the self-consistency of the prior belief that agents (believe they) will choose free-energy-minimizing policies, nor the full derivation of the expected free energy here. Interested readers can find the full derivations in Friston et al. (2015, 2017a) and Parr and Friston (2019). However, it is worth emphasizing that different components of the expected free energy clarify its implications for optimal behavior in active inference agents. These components are formally related to other discussions of adaptive behavior, such as the trade-off between exploration and exploitation. We can re-write the expected free energy for a given time-point τ and policy π as a bound on the sum of two expectations:

(8) $$\begin{array}{l} G(\tau ,\pi )={𝔼}_{Q(o_{\tau },s_{\tau }|\pi )}[\text{ln }Q(s_{\tau }|\pi )-\text{ln }P(o_{\tau },s_{\tau })]\\ \;\geq -{𝔼}_{Q(o_{\tau }|\pi )}[{\text{D}}_{KL}[Q(s_{\tau }|o_{\tau },\pi )||Q(s_{\tau }|\pi )]]\\ \text{ ; }-{𝔼}_{Q(o_{\tau }|\pi )}[\text{ln }P(o_{\tau })] \end{array}$$

From this decomposition of the quantity bounded by the expected free energy *G* we illustrate the different kinds of “value” that contribute to behavior in active inference (Friston et al., 2013, 2015; Parr and Friston, 2017; Mirza et al., 2018). See the Appendix for a derivation of Equation (8). The left term on the RHS of the second line is a term that has been called *negative information gain*. Under active inference, the most likely policies are those that *minimize* the expected free energy of their sensory consequences—therefore, minimizing this left term promotes policies that disclose information about the environment by reducing uncertainty about the causes of observations, i.e., maximizing information gain. The right term on the RHS of the second line is often called negative extrinsic (or instrumental) value, and minimizing this term promotes policies that lead to observations that match the agent's prior expectations about observations, The relationship of these prior expectations to goal-directed behavior will become clear later in this section. We also offer an alternative decomposition of the expected free energy, formulating it in terms of minimizing a combination of *ambiguity* and *risk*:

(9) $$\begin{array}{l} \text{G}(\tau ,\pi )\geq -\underset{Epistemic\;value}{\underset{︸}{{\text{E}}_{Q(o_{\tau }|\pi )}[{\text{D}}_{KL}[Q(s_{\tau }|o_{\tau },\pi )\|Q(s_{\tau }|\pi )]]}}\\ \;-\underset{Instrumental\;value}{\underset{︸}{{\text{E}}_{Q(o_{\tau }|\pi )}[\text{ln }P(o_{\tau })]}}]\\ \;=\underset{Ambiguity}{\underset{︸}{{𝔼}_{Q(s_{\tau }|\pi )}[\text{H}[P(o_{\tau }|s_{\tau })]]}}+\underset{Risk}{\underset{︸}{{\text{D}}_{KL}[Q(o_{\tau }|\pi )||P(o_{\tau })]}} \end{array}$$

See the Appendix for a derivation of Equation (9). The first term on the RHS of the first line (previously referred to as information gain) we hereafter refer to as “epistemic value” (Friston et al., 2015; Mirza et al., 2016). It is equivalent to expected Bayesian surprise in other accounts of information-seeking behavior and curiosity (Linsker, 1990; Itti and Baldi, 2009; Gottlieb and Oudeyer, 2018). Such an epistemic drive has the effect of promoting actions that uncover information about hidden states via sampling informative observations. This intrinsic drive to uncover information, and its natural emergence via the minimization of expected free energy, is integral to accounts of exploratory behavior, curiosity, salience, and related active-sensing phenomena under active inference (FitzGerald et al., 2015; Friston et al., 2017b,d; Parr and Friston, 2017, 2018b; Mirza et al., 2019b). An alternative formulation of the expected free energy is given in the second line of Equation (9), where minimizing expected free energy promotes policies that reduce “ambiguity,” defined as the expected uncertainty of observations, given the states expected under a policy. These notions of information gain and expected uncertainty will serve as a useful construct in understanding the behavior of active inference agents performing hierarchical scene construction later.

In order to understand how minimizing expected free energy **G** relates to the pursuit of preference-related goals or drives, we now turn to the second term on the RHS of the first line of Equation (9). In order to enable instrumental or “non-epistemic” goals to drive action, we supplement the agent's generative model with an unconditional distribution over observations *P*(*o*) (sometimes called *P*(*o*|*m*), where *m* indicates conditioning on the generative model of the agent)—this also factors into the log joint probability distribution in the first line of Equation (8). By fixing certain outcomes to have high (or low) probabilities as prior beliefs, minimizing **G** imbues action selection with an apparent instrumental or exploitative component, measured by how closely observations expected under a policy align with baseline expectations. Said differently: active inference agents pursue policies that result in outcomes that they *a priori* expect to encounter. The distribution *P*(*o*) is therefore also often called the “prior preferences.” Encoding preferences or desires as beliefs about future sensory outcomes underwrites the known duality between inference and optimal control (Todorov, 2008; Friston et al., 2009; Friston, 2011; Millidge et al., 2020). In the language of Expected Utility Theory (which explains behavior by appealing to the principle of maximizing expected rewards), the logarithm of such prior beliefs is equivalent to the utility function (Zeki et al., 2004). This component of **G** has variously been referred to as utility, extrinsic value, or instrumental value (Seth, 2015; Friston et al., 2017a; Biehl et al., 2018; Seth and Tsakiris, 2018); hereafter we will use the term instrumental value. A related but subtly different perspective is provided by the second term on the RHS of the second line of Equation (9): in this formulation, prior preferences enter the free energy through a “risk” term. The minimization of expected risk favors actions that minimize the KL-divergence between outcomes expected under a policy and preferred outcomes, and is related to formulations like KL-control or risk-sensitive control (Klyubin et al., 2005; van den Broek et al., 2010).

## 3. Scene Construction With Random Dot Motion

### 3.1. The Original Model

**Figure 1:**
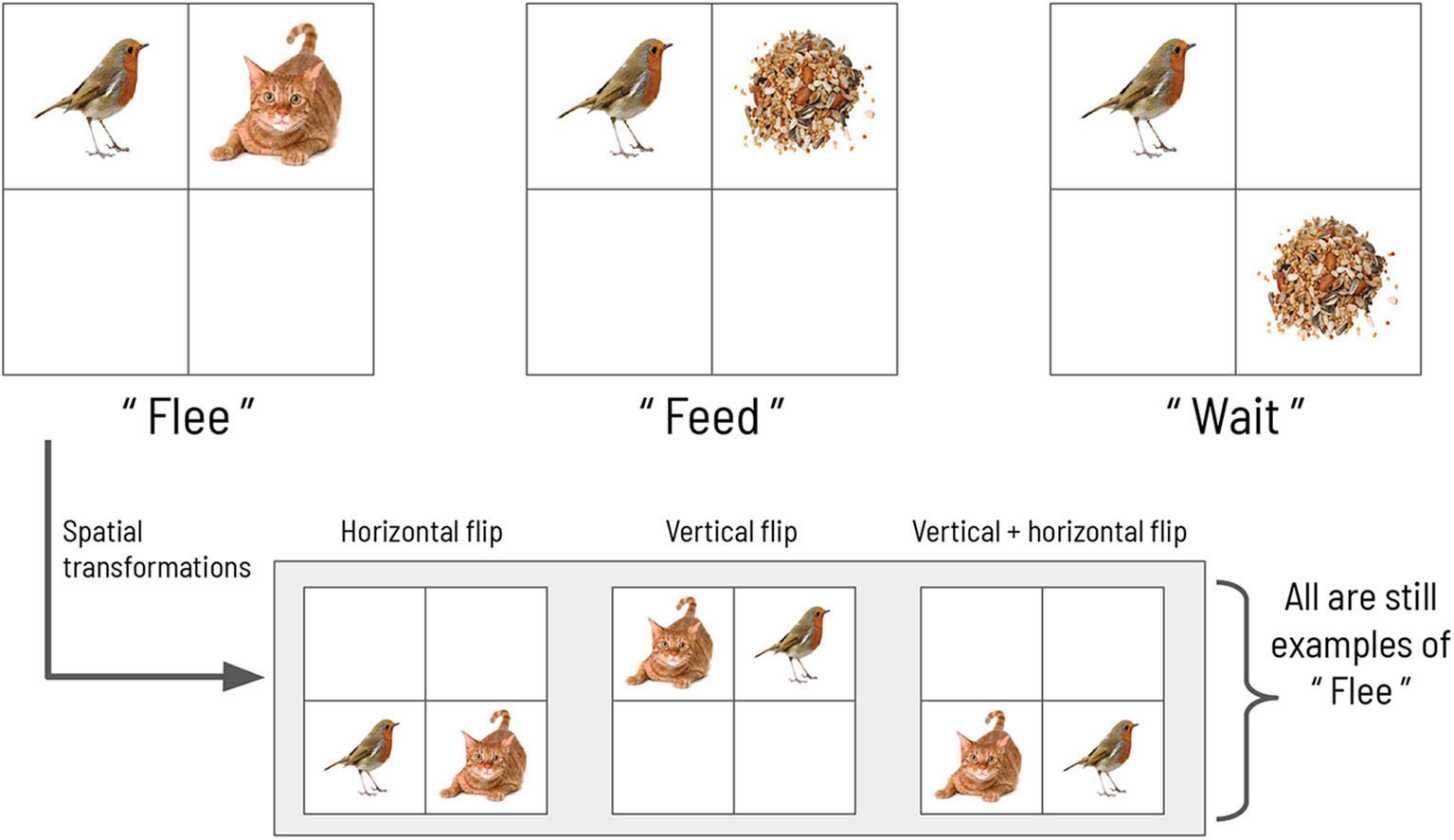
The scene configurations of the original formulation. The three scenes characterizing each trial in the original scene construction study, adapted with permission from Mirza et al. (2016).

We now describe an abstract scene construction task that will serve as the experimental context within which to frame our hierarchical account of active evidence accumulation. Inspired by a previous active inference model of scene construction introduced by Mirza et al. (2016), here we invoke scene construction in service of a categorization game. In each trial of the task, the agent must make a discrete choice to report its belief about the identity of the “hidden scene.” In the formulation by Mirza et al., the scenes are represented by three abstract semantic labels: “flee,” “feed,” and “wait” (see Figure 1). Each scene manifests as a particular spatial coincidence of two pictures, where each picture is found within a single quadrant in a 2 × 2 visual array. For example, the “flee” scene is defined as when a picture of a cat and a picture of a bird occupy two quadrants lying horizontally adjacent to one other. The scene identities are also invariant to two spatial transformations: vertical and horizontal inversions. For example, in the “flee” scene, the bird and cat pictures can be found in either in the top or bottom row of the 2 × 2 array, and they can swap positions; in any of these cases the scene is still “flee.” The task requires active visual interrogation of the environment because quadrants must be *gaze-contingently* unveiled. That means, by default all quadrants are covered and their contents not visible; the agent must directly look at a quadrant in order to see its contents. This task structure and the ambiguous nature of the picture → scene mapping means that agents need to actively forage for information in the visual array in order to abduce the scene.

### 3.2. Introducing Random Dot Motion

**Figure 2:**
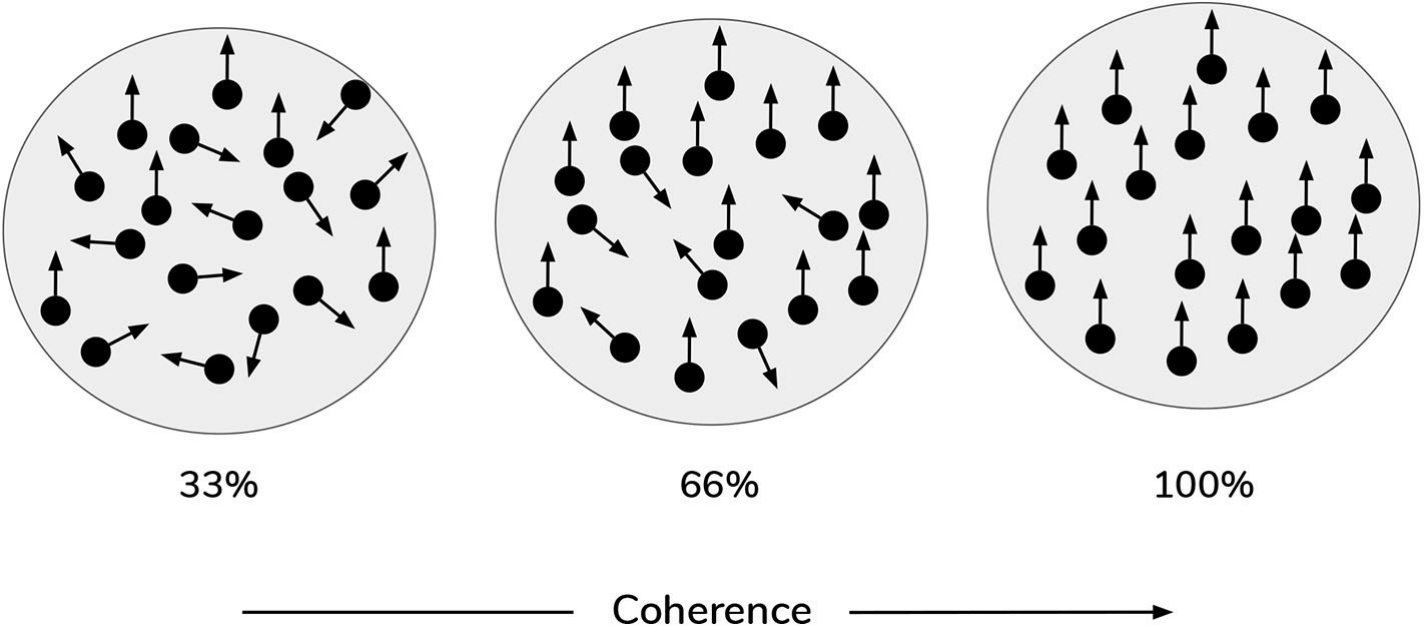
Random Dot Motion Stimuli (RDMs). Schematic of random dot motion stimuli, with increasing coherence levels (i.e., % percentage of dots moving upwards) from left to right.

In the current work, scene construction is also framed as a categorization task, requiring the gaze-contingent disclosure of quadrants whose contents furnish evidence for beliefs about the scene identity. However, in the new task, the visual stimuli occupying the quadrants are animated *random dot motion* or RDM patterns, instead of static pictographs. An RDM stimulus consists of a small patch of dots whose correlated displacement over time gives rise to the perception of apparent directed motion (see Figure 2). By manipulating the proportion of dots moving in the same direction, the apparent direction of motion can be made more or less difficult to discriminate (Shadlen and Newsome, 1996). This discriminability is usually operationalized as a single *coherence* parameter, which defines the percentage of dots that appear to move in a common direction. The remaining non-signal (or “incoherent”) dots are usually designed to move in random independent directions. This coherence parameter thus becomes a simple proxy for sensory uncertainty in motion perception: manipulating the coherence of RDM patterns has well-documented effects on behavioral measures of performance, such as reaction time and discrimination accuracy, with increasing coherence leading to faster reaction times and higher accuracy (Palmer et al., 2005). In the current formulation, each RDM pattern is characterized by a unique primary direction of motion that belongs to one of the four cardinal directions: **UP**, **RIGHT**, **DOWN**, or **LEFT**. For example, in a given trial one quadrant may contain a motion pattern moving (on average) upwards, while another quadrant contains a motion pattern moving (on average) leftwards. These RDM stimuli are suitable for the current task because we can use the coherence parameter to tune motion ambiguity and hence sensory uncertainty. Applying this metaphor to the original task (Mirza et al., 2016): we might imagine blurred versions of the cat and bird pictures, such that it becomes difficult to tell whether a given image is of a bird or a cat—this low-level uncertainty about individual images may then “carry forward” to affect scene inference. An equivalent analogy might be found in the problem of reading a hastily-written phone number, such that it becomes hard to distinguish the number “7” from the number “1.” In our case, the motion coherence of RDMs controls how easily an RDM of one direction can be confused with another direction—namely, a more incoherent dot pattern is more likely to be mistaken as a dot pattern moving in a different direction.

**Figure 3:**
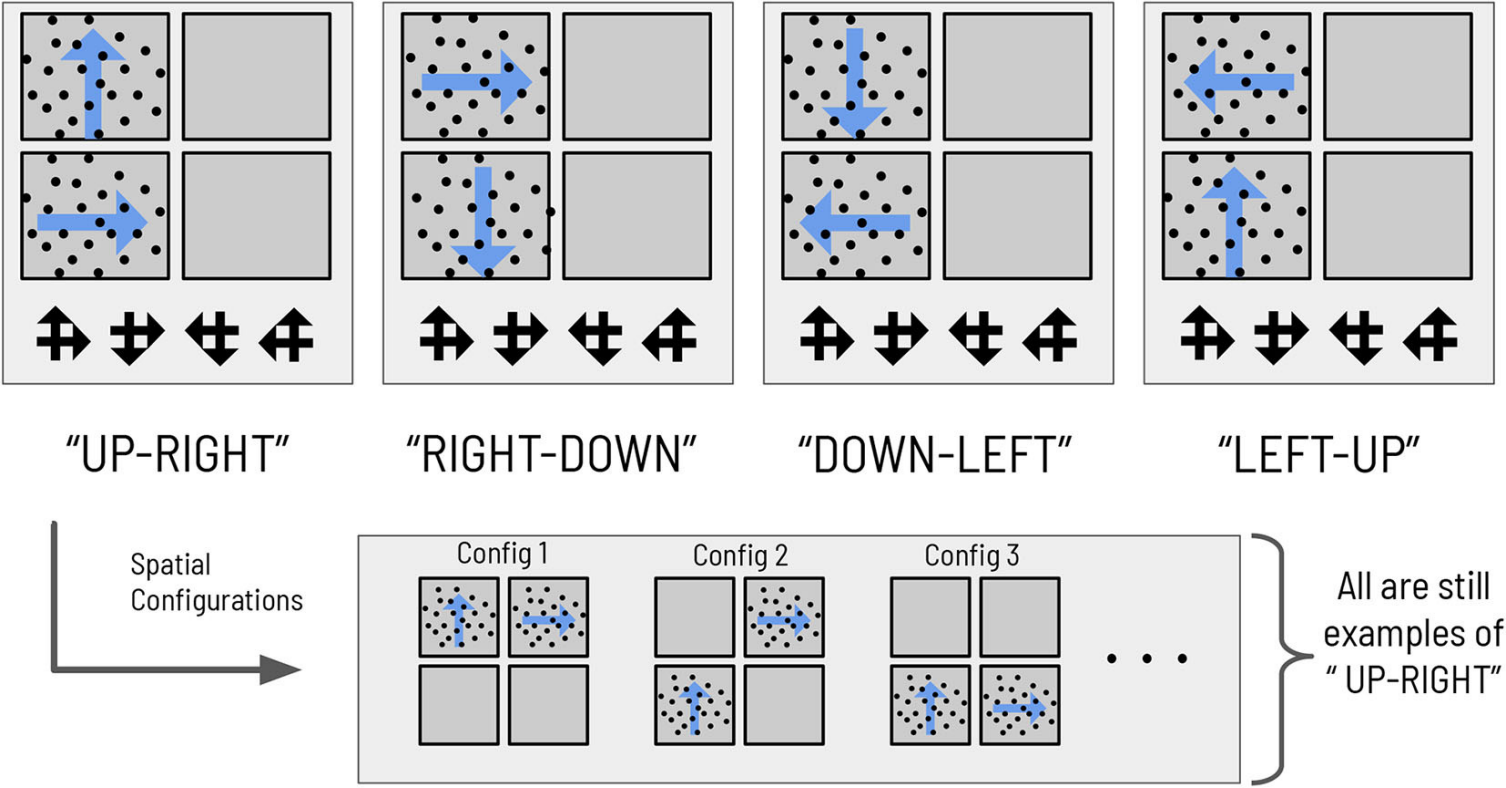
The mapping between scenes and RDMs. The mapping between the four abstract scene categories and their respective dot motion pattern manifestations in the context of the hierarchical scene construction task. As an example of the spatial invariance of each scene, the bottom right panels show two possible (out of 12 total) RDM configurations for the scene “**RIGHT-DOWN**,” where the two constitutive RDMs of that scene are found in exactly two of the four quadrants. The ‘scene symbols' at the bottom of the visual array represent the categorization choices available to the subject, with each symbol comprised of two overlapping arrows that indicate the directions of the motion that define the scene.

We also design the visual stimulus → scene mapping such that scenes are degenerate with respect to individual visual stimuli, as in the previous task (see Figure 3). There are four scenes, each one defined as the co-occurrence of two RDMs in two (and only two) quadrants of the visual array. The two RDMs defining a given scene move in perpendicular directions; the scenes are hence named: **UP-RIGHT**, **RIGHT-DOWN**, **DOWN-LEFT**, and **LEFT-UP**. Discerning the direction of one RDM is not sufficient to disambiguate the scene; due to the degeneracy of the scene configurations with respect to RDMs, the agent must always observe two RDMs and discern their respective directions before being able to unambiguously infer scene identity. The task requires two nested inferences—one about the contents of the currently-fixated quadrant (e.g., “Am I looking at an **UP**-wards moving RDM?”) and another about the identity about the overarching scene (e.g., “is the scene **UP-RIGHT**?”). During each trial, an agent can report its guess about the scene identity by choosing one of the four symbols that signify the scenes (see Figure 3), which ends the trial. This concludes our narrative description of the experimental setup.

### 3.3. Summary

We have seen how both perception and action emerge as consequences of free energy minimization under active inference. Perception is analogized to state estimation and corresponds to optimizing variational beliefs about the hidden causes of sensory data *x*. Meanwhile actions are sampled from inferred sequences of control states (policies). The likelihood of a policy is inversely proportional to the free energy *expected* under that policy. We demonstrated that expected free energy can be decomposed into the sum of two terms, which respectively encode the drive to resolve ambiguity about the hidden causes of sensory data (epistemic value) and to satisfy agent-specific preferences (instrumental value) (first line of Equation 9). In this way active inference theoretically dissolves the exploration-exploitation dilemma often discussed in decision sciences and reinforcement learning (March, 1991; Schmidhuber, 1991; Sutton and Barto, 1998; Parr, 2020) by choosing policies that minimize expected free energy. This unification of perception and action under a common Bayesian ontology underlies the power of active inference as a normative framework for studying adaptive behavior in complex systems. In the following sections we will present a (hierarchical) Markov Decision Process model of scene construction, where stochastic motion stimuli serve as observations for an overarching scene categorization task. We then discuss perception and action under active inference in the context of hierarchical scene construction, with accompanying computational demonstrations.

## 4. Hierarchical Markov Decision Process for Scene Construction

We now introduce the hierarchical active inference model of visual foraging and scene construction. The generative model (the agent) and the generative process of the environment both take the form of a Markov Decision Process or MDP. MDPs are a simple class of probabilistic generative models where space and time are treated discretely (Puterman, 1995). In the MDP used here, states are treated as discrete samples from categorical distributions and likelihoods act as linear transformations of hidden states, mapping states at one time step to the subsequent time step, i.e., *P*(*s*_*t*_|*s*_*t*−1_). This specification imbues the environment with Markovian, or “memoryless” dynamics. An extension of the standard MDP formulation is the *partially-observed* MDP or POMDP, which includes discrete observations that are mapped (via a likelihood function *P*(*o*_*t*_|*s*_*t*_)) from states to observations at a given time.

A generative model is simply a joint probability distribution over sensory observations and their latent causes *P*(õ, *x*), and is often factorized into the product of a likelihood and a set of marginal distributions over latent variables and hyperparameters, e.g., $P\left(õ|\tilde{s}\right)P\left(\tilde{s}\right)P\left(\varphi \right)P\left(\zeta \right)\ldots$ where $\tilde{s},\varphi ,\zeta ,\ldots \in x$ refer to the various latent causes. Note that in the current formulation the only hidden variables subject to variational inference are hidden states $\tilde{s}$ and policies π. The discrete MDP constrains these distributions to have a particular form; here, the priors over initial states, transition and likelihood matrices are encoded as categorical distributions over a discrete set of states and observations. Agents can only directly observe sensory outcomes õ, meaning that the agent must infer hidden states $\tilde{s}$ by inverting the generative model to estimate the causes of observations. Hierarchical models take this a step further by adding multiple layers of hidden-state inference, allowing beliefs about hidden states ${\tilde{s}}^{\left(i\right)}$ at one level to act as so-called “inferred observations” õ^(*i*+1)^ for the level above, with associated priors and likelihoods operating at all levels. This marks a departure from previous work in the hierarchical POMDP literature (Pineau et al., 2001; Theocharous et al., 2004), where the hierarchical decomposition of *action* is emphasized and used to finesse the exponential costs of planning; states and observations, on the other hand, are often coarse-grained using separate schemes or left fully enumerated (although see Sridharan et al., 2010). In the current formulation, we adopt a hybrid scheme, where at a given level of depth in the hierarchy, observations can be *both* passed in at the same level (from the generative process), as well as via “inferred” observations from the level below. Note that as with õ, we use $\tilde{s}$ to denote a sequence of hidden states over time $\tilde{s}={\left[s_{1},s_{2},\ldots s_{T}\right]}^{T}$.

### 4.1. Hierarchical MDPs

**Figure 4:**
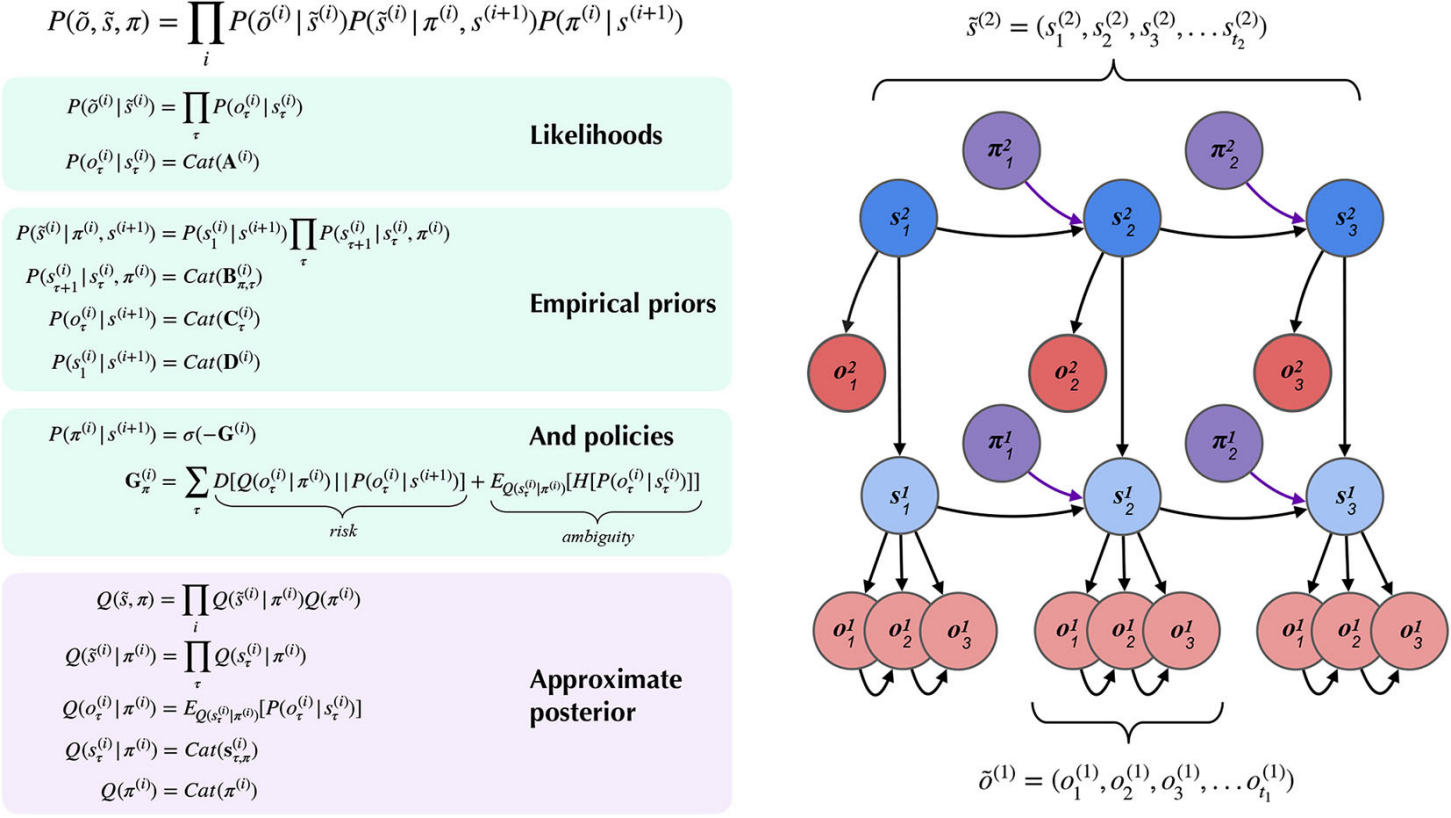
A partially-observed Markov Decision Process with two hierarchical layers. Schematic overview of the generative model for a hierarchical partially-observed Markov Decision Process. The generic forms of the likelihoods, priors, and posteriors at hierarchical levels are provided in the left panels, adapted with permission from Friston et al. (2017d). *Cat*(**x**) indicates a categorical distribution, and $\tilde{x}$ indicates a discrete sequence of states or random variables: $\tilde{x}=\left(x_{1},x_{2},\ldots ,x_{t}\right)$. Note that priors at the highest level (Level 2) are not shown, but are unconditional (non-empirical) priors, and their particular forms for the scene construction task are described in the text. As shown in the “Empirical Priors” panel, prior preferences at lower levels ${\text{C}}_{\tau }^{\left(i\right)}$ can be a function of states at level *i* + 1, but this conditioning of preferences is not necessary, and in the current work we pre-determine prior preferences at lower levels, i.e., they are not contextualized by states at higher levels (see Figure 8). Posterior beliefs about policies are given by a softmax function of the expected free energy of policies at a given level. The approximate (variational) beliefs over hidden states are represented via a mean-field approximation of the full posterior, such that hidden states can be encoded as the product of marginal distributions. Factorization of the posterior is assumed across hierarchical layers, across hidden state factors (see the text and Figures 6, 7 for details on the meanings of different factors), and across time. “Observations” at the higher level (õ^(2)^) may belong to one of two types: (1) observations that directly parameterize hidden states at the lower level via the composition of the observation likelihood one level *P*(*o*^(*i* + 1)^|*s*^(*i* + 1)^) with the empirical prior or “link function” *P*(*s*^(*i*)^|*o*^(*i* + 1)^) at the level below, and (2) observations that are directly sampled *at the same level* from the generative process (and accompanying likelihood of the generative model *P*(*o*^(*i* + 1)^|*s*^(*i* + 1)^)). For conciseness, we represent the first type of mapping, from states at *i* + 1 to states at *i* through a direct dependency in the Bayesian graphical model in the right panel, but the reader should note that in practice this is achieved via the composition of two likelihoods: the observation likelihood at level *i* + 1 and the link function at level *i*. This composition is represented by a single empirical prior *P*(*s*^(*i*)^|*s*^(*i* + 1)^) = *Cat*(**D**^(*i*)^) in the left panel. In contrast, all observations at the lowest level (õ^(1)^) feed directly from the generative process to the agent.

Figure 4 summarizes the structure of a generic two-layer hierarchical POMDP model, outlining relationships between random variables via a Bayesian graph and their (factorized, categorical) forms in the left panel. In the left panel of Figure 4, õ and $\tilde{s}$ indicate sequences of observations and states over time. In the MDP model, the probability distributions that involve these sequences are expressed in a factorized fashion. The model's beliefs about how hidden states ${\tilde{s}}^{\left(i\right)}$ cause observations õ^(*i*)^ are expressed as multidimensional arrays in the likelihood matrix **A**^(*i*),*m*^, where *i* indicates the index of the hierarchical level and *m* indicates a particular modality (Mirza et al., 2016; Friston et al., 2017d). The (*x, y*) entry of a likelihood matrix **A**^(*i*),*m*^ prescribes the probability of observing the outcome *x* under the modality *m* at level *i*, given hidden state *y*. In this way, the columns of the **A** matrices are conditional categorical distributions over outcomes, given the hidden state indexed by the column. The dynamics that describe how hidden states at a given level *s*^(*i*)^ evolve over time are given by Markov transition matrices **B**^(*i*),*n*^(*u*) which express how likely the next state is given the current state—in the generative model, this is equivalent to the transition distribution *P*(*s*_*t*_|*s*_*t*−1_, *u*_*t*_). Here *n* indexes a particular factor of level *i*'s hidden states, and *u* indexes a particular control state or action. Actions in this scheme are thus treated as controlled transitions between hidden states. We assume that the posterior distribution over different dimensions of hidden states factorize, leading to conditional independence between separate hidden state factors. This is known as the *mean-field approximation*, and allows the sufficient statistics of posterior beliefs about different hidden state variables to be updated separately (Feynman, 1998). This results in a set of relatively simple update equations for posterior beliefs and is also consistent with known features of neuroanatomy, e.g., functional segregation in the brain (Felleman and Van, 1991; Ungerleider and Haxby, 1994; Friston and Buzsáki, 2016; Mirza et al., 2016; Parr and Friston, 2018a). The hierarchical MDP formulation notably permits a segregation of timescales across layers and an according mean-field approximation on their respective free energies, such that multiple time steps of belief-updating at one level can unfold within a single time step of inference at the level above. In this way, low-level beliefs about hidden states (and policies) can be accumulated over time at a lower layer, at which point the final posterior estimate about hidden states is passed “up” as an *inferred* outcome to the layer above. Subsequent layers proceed at their own characteristic (slower) timescales (Friston et al., 2017d) to update beliefs about hidden states at their respective levels. Before we describe the particular form of the hierarchical MDP used (as both the generative process and generative model) for deep scene construction, we provide a brief technical overview of the update scheme used to solve POMDPs with active inference.

#### 4.1.1. Belief Updating

**Figure 5:**
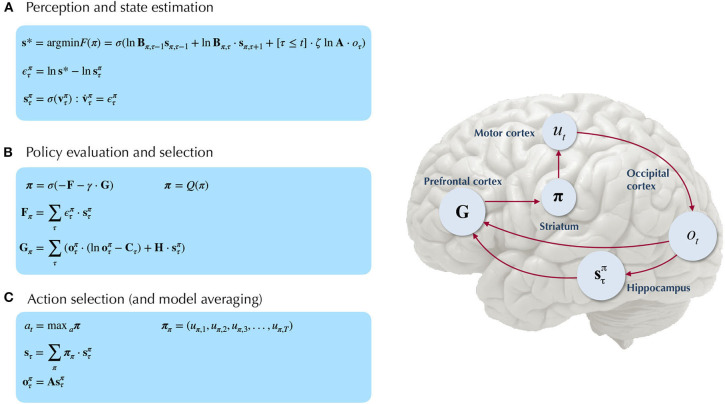
Belief-updating under active inference. Overview of the update equations for posterior beliefs under active inference. **(A)** Shows the optimal solution for posterior beliefs about hidden states s^*^ that minimizes the variational free energy of observations. In practice the variational posterior over states is computed as a marginal message passing routine (Parr et al., 2019), where prediction errors $\varepsilon_{\tau }^{\pi }$ minimized over time until some criterion of convergence is reached (ε ≈ 0). The prediction errors measure the difference between the current log posterior over states $\ln\;{\text{s}}_{\tau }^{\pi }$ and the optimal solution ln s^*^. Solving via error-minimization lends the scheme a degree of biological plausibility and is consistent with process theories of neural function like predictive coding (Bastos et al., 2012; Bogacz, 2017). An alternative scheme would be equating the marginal posterior over hidden states (for a given factor and/or timestep) to the optimal solution ${\text{s}}_{\pi ,\tau }^{*}$—this is achieved by solving for s^*^ when free energy is at its minimum (for a particular marginal), i.e., $\frac{\partial F}{\partial {\text{s}}_{\pi ,\tau }}=0$. This corresponds to a fixed-point minimization scheme (also known as coordinate-ascent iteration), where each conditional marginal is iteratively fixed to its free-energy minimum, while holding the remaining marginals constant (Blei et al., 2017). **(B)** Shows how posterior beliefs about policies are a function of the free energy of states expected under policies **F** and the expected free energy of policies **G**. **F** is a function of state prediction errors and expected states, and **G** is the expected free energy of observations under policies, shown here decomposed into the KL divergence between expected and preferred observations or risk (${\text{o}}_{\tau }^{\pi }\cdot \left(\ln{\text{o}}_{\tau }^{\pi }-{\text{C}}_{\tau }\right)$) and the expected entropy or ambiguity ($\text{H}\cdot {\text{s}}_{\tau }^{\pi }$). A precision parameter γ scales the expected free energy and serves as an inverse temperature parameter for a softmax normalization σ of policies. See the text (Section 4.1.1) for more clarification on the free energy of policies **F**. **(C)** Shows how actions are sampled from the posterior over policies, and the posterior over states is updated via a Bayesian model average, where expected states are averaged under beliefs about policies. Finally, expected observations are computed by passing expected states through the likelihood of the generative model. The right side shows a plausible correspondence between several key variables in an MDP generative model and known neuroanatomy. For simplicity, a hierarchical generative model is not shown here, but one can easily imagine a hierarchy of state inference that characterizes the recurrent message passing between lower-level occipital areas (e.g., primary visual cortex) through higher level visual cortical areas, and terminating in “high-level,” prospective and policy-conditioned state estimation in areas like the hippocampus. We note that it is an open empirical question, whether various computations required for active inference can be localized to different functional brain areas. This figure suggests a simple scheme that attributes different computations to segregated brain areas, based on their known function and neuroanatomy (e.g., computing the expected free energy of actions (G), speculated to occur in frontal areas).

Figure 5 provides a schematic overview of the belief update equations for state estimation and policy inference under active inference. For the sake of clarity here we only consider a single “layer” of a POMDP generative model, i.e., we don't include the top-down or bottom-up beliefs that parameterize priors over hidden states (from the layer above) or inferred observations (from the layer below). Note that in this formulation, instead of directly evaluating the solution for states with lowest free energy s^*^, we use a marginal message passing routine to perform a gradient descent on the variational free energy at each time step, where posterior beliefs about hidden states and policies are incremented using prediction errors ε (see Figure 5 legend for more details). In the context of deep temporal models, these equations proceed independently at each level of the hierarchy at each time step. At lower levels, the posterior over certain hidden state factors at the first timestep ${\text{s}}_{1}^{\left(i\right)}$ can be initialized as the “expected observations” **o**^(*i*+1)^ from the level above, and “inferred observations” at higher levels are inherited as the final posterior beliefs ${\text{s}}_{T}^{\left(i\right)}$ over the corresponding hidden state at lower levels. This update scheme may sound complicated; however, when expressed as a gradient descent on free energy, with respect to the sufficient statistics of beliefs about expected states, it reduces to a remarkably simple scheme that bears resemblance to neuronal processing: see Friston et al. (2015) for details. Importantly, the mean-field factorization of the generative model across hierarchical layers allows the belief updating to occur in isolation at each layer of the hierarchy, such that only the final posterior beliefs at one layer need to be passed to the layer above. The right side of Figure 5 shows a simple schematic of how the particular random variables that make up generative model might correspond to neural processing in known brain regions. Evidence for the sort of hierarchical processing entailed by such generative models abounds in the brain, and is the subject of a wealth of empirical and theoretical neuroscience research (Lee and Mumford, 2003; Friston, 2008; Hasson et al., 2008; Friston et al., 2017c; Runyan et al., 2017; Pezzulo et al., 2018).

We also find it worthwhile to clarify the distinction between the *variational free energy* of policies *F*(π) and the *expected free energy* of policies *G*(π), both of which are needed to compute the posterior over policies *Q*(π). The final posterior probability over policies is a softmax function of both quantities (see Figure 5B), where the former can be seen as the evidence afforded by past and ongoing observations, that a given policy is *currently* being pursued, whereas the latter is the evidence *expected* to be gathered in favor of pursuing a given policy, where this expected evidence is biased by prior beliefs about what kinds of observations the agent is likely to encounter (via the prior preferences **C**). Starting with the definition of the free energy of the (approximate) posterior over both hidden states and policies::

(10) $$\begin{array}{l} F={\text{E}}_{Q(\tilde{s},\pi )}[\text{ln }Q(\tilde{s},\pi )-\text{ln }P(\tilde{o},\tilde{s},\pi )]\\ \;={\text{E}}_{Q(\pi )}[F(\pi )]+{\text{D}}_{KL}[Q(\pi )||P(\pi )] \end{array}$$

(11) $$\begin{array}{l} F(\pi )=-{\text{E}}_{Q(\tilde{s}|\pi )}\left[\text{ln }P(\tilde{o},\tilde{s}|\pi )\right]-\text{H}[Q(\tilde{s}|\pi )]\\ Q(\pi )=\text{arg }\underset{Q(\pi )}{\text{min }}F\propto e^{\left(\text{ln }P(\pi )-F(\pi )\right)} \end{array}$$

Where ln *P*(π) = *G*(π) is a prior of the generative model that encodes the self-consistent belief that the prior probability of a policy is proportional to its negative expected free energy *G*(π). Please see the Appendix for a fuller derivation of Equation (10). Note that (due to the factorization of the approximate posterior over time, cf. Section 2.2) the variational free energy of a policy *F*(π) is the sum of the individual free energies for a given policy afforded by past observations, up to and including the current observation:

(12) $$\begin{array}{l} F(\pi )=\sum_{\tau }F(\pi ,\tau )\\ F(\pi ,\tau )=-{\text{E}}_{Q(s_{\tau }|\pi )Q(s_{\tau -1}|\pi )}[\left[\tau \leq t]\text{ln }P(o_{\tau }|s_{\tau }\right)\\ \;+\text{ln }P(s_{\tau }|s_{\tau -1},\pi )-\text{ln }Q(s_{\tau }|\pi )] \end{array}$$

The Iverson brackets [τ ≤ *t*] return 1 if τ ≤ *t* and 0 otherwise.

### 4.2. From Motion Discrimination to Scene Construction: A Nested Inference Problem

**Figure 6:**
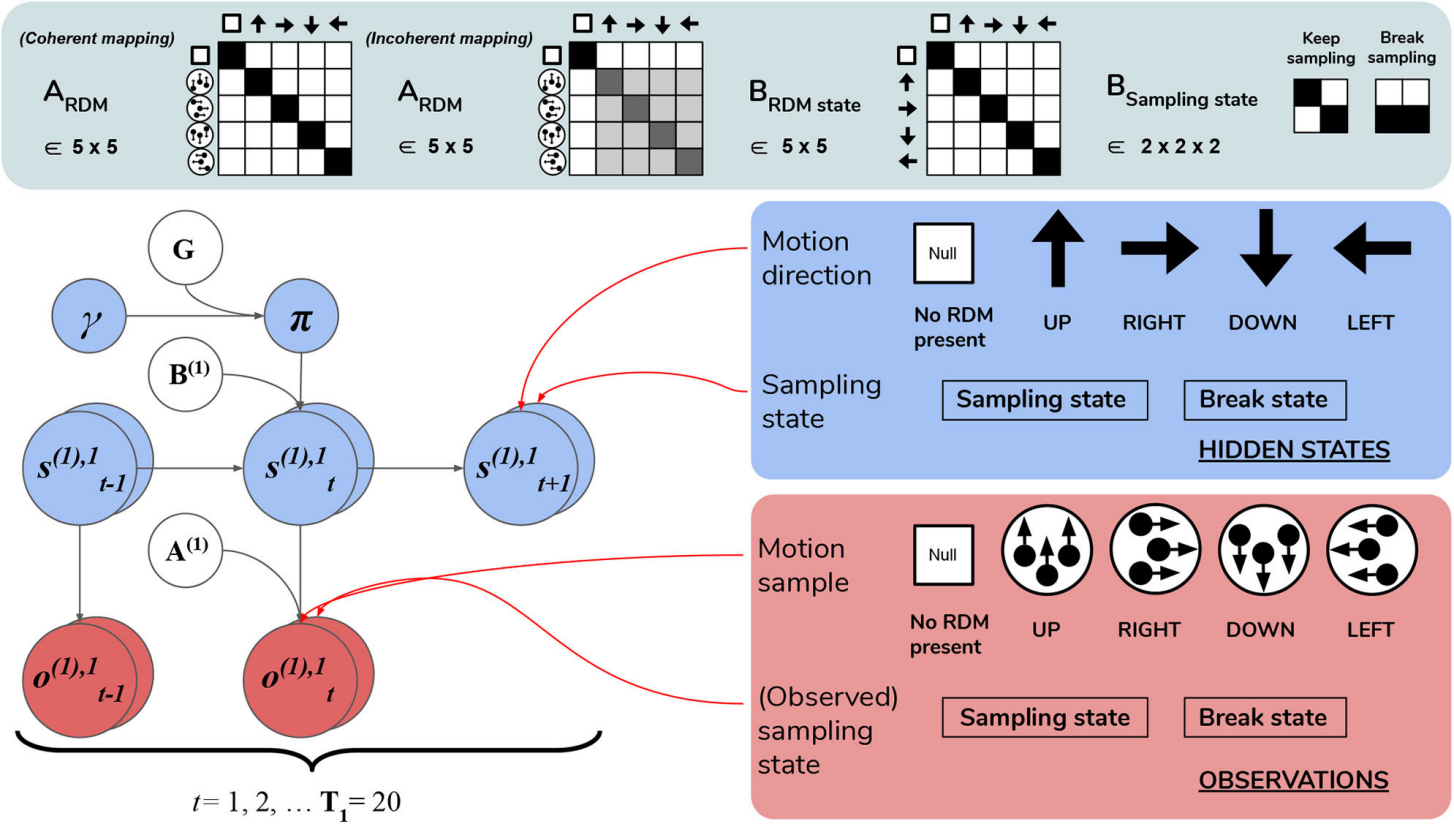
Level 1 MDP. **Level 1** of the hierarchical POMDP for scene construction (see Section 4.2.1 for details). Level 1 directly interfaces with stochastic motion observations generated by the environment. At this level hidden states correspond to: (1) the true motion direction **s**^(1),1^ underlying visual observations at the currently-fixated region of the visual array and (2) the sampling state **s**^(1),2^, an aspect of the environment that can be changed via actions, i.e., selections of the appropriate state transition, as encoded in the **B** matrix. The first hidden state factor **s**^(1),1^ can either correspond to a state with no motion signal (“Null,” in the case when there is no RDM or a categorization decision is being made) or assume one of the four discrete values corresponding to the four cardinal motion directions. At each time step of the generative process, the current state of the RDM stimulus **s**^(1),1^ is probabilistically mapped to a motion observation via the first-factor likelihood **A**^(1),1^ (shown in the top panel as **A**_RDM_). The entropy of the columns of this mapping can be used to parameterize the coherence of the RDM stimulus, such that the true motion states **s**^(1),1^ cause motion observations **o**^(1),1^ with varying degrees of fidelity. This is demonstrated by two exemplary **A**_RDM state_ matrices in the top panel (these correspond to **A**^(1),1^): the left-most matrix shows a noiseless, “coherent” mapping, analogized to the situation of when an RDM consists of all dots moving in the same direction as described by the true hidden state; the matrix to the right of the noiseless mapping corresponds to an incoherent RDM, where instantaneous motion observations may assume directions different than the true motion direction state, with the frequency of this deviation encoded by probabilities stored in the corresponding column of **A**_RDM_. The motion direction state doesn't change in the course of a trial (see the identity matrix shown in the top panel as **B**_RDM_, which simply maps the hidden state to itself at each subsequent time step)—this is true of both the generative model and the generative process. The second hidden state factor **s**^(1),2^ encodes the current “sampling state” of the agent; there are two levels under this factor: “**Keep-sampling**” or “**Break-sampling**.” This sampling state (a factor of the generative process) is directly represented as a control state in the generative model; namely, the agent can change it by sampling actions (B-matrix transitions) from the posterior beliefs about policies. The agent believes that the “**Break-sampling**” state is a *sink* in the transition dynamics, such that once it is entered, it cannot be exited (see the right-most matrix of the transition likelihood **B**_Sampling state_). Entering the “**Break-sampling**” state terminates the POMDP at Level 1. The “**Keep-sampling**” state enables the continued generation of motion observations as samples from the likelihood mapping **A**^(1),1^. **A**^(1),2^ (the “proprioceptive” likelihood, not shown for clarity) deterministically maps the current sampling state **s**^(1),2^ to an observation **o**^(1),2^ thereof (bottom row of lower right panel), so that the agent always observes which sampling state it is in unambiguously.

We now introduce the deep, temporal model of scene construction using the task discussed in Section 3 as our example (Figure 6). We formulate perception and action with a hierarchical POMDP consisting of two distinct layers that are solved via active inference. The first, shallowest level (Level 1) is an MDP that updates posterior beliefs about the most likely cause of visual stimulation (RDM direction), where we model the ongoing contents of single fixations—the stationary periods of relative retinal-stability between saccadic eye movements. This inference is achieved with respect to the (spatially-local) visual stimuli underlying current foveal observations. A binary policy is also implemented, encoding the option to continue holding fixation (and thus keep sampling the current stimulus) or to interrupt sampling and terminate updates at the lower level. The second, higher level (Level 2) is another MDP that performs inference at a slower timescale, with respect to the overarching hidden scene that describes the current trial. Here, we enable policies that realize visual foraging. These policies encode controlled transitions between different states of the oculomotor system, serving as a model of saccadic eye movements to different parts of the visual array. This method of encoding saccades as controlled transitions between locations is inspired by the original scene construction formulation in Mirza et al. (2016). We will now discuss both layers individually and translate different elements of the MDP generative model and environment to task-relevant parameters and the beliefs of the agent.

#### 4.2.1. Level 1: Motion Discrimination via Motion Sampling Over Time

Lowest level (Level 1) beliefs are updated as the agent encounters a stream of ongoing, potentially ambiguous visual observations—the instantaneous contents of an individual fixation. The hidden states at this level describe a distribution over motion directions, which parameterize the true state of the random motion stimulus within the currently-fixated quadrant. Observations manifest as a sequence of stochastic motion signals that are samples from the true hidden state distribution.

The generative model has an identical form as the generative process (see above) used to generate the stream of Level 1 outcomes. Namely, it is comprised of a set of likelihoods and transitions as the dynamics describing the “real” environment (Figure 6). In order to generate a motion observation, we sample the probability distribution over motion direction given the true hidden state using the Level 1 generative process likelihood matrix **A**^(1),1^. For example, if the current true hidden state at the lower level is **2** (implying that an RDM stimulus of **UP**wards motion occupies the currently fixated quadrant), stochastic motion observations are sampled from the *second* column of the generative likelihood mapping **A**^(1),1^. The precision of this column-encoded distribution over motion observations determines how often the sampled motions will be **UP**wards signals and thus consistent with the true hidden state. The entropy or ambiguity of this likelihood mapping operationalizes sensory uncertainty and in this case, motion incoherence. For more details on how states and outcomes are discretized in the generative process, see Figure 6 and its legend.

Inference about the motion direction (Level 1 state estimation) roughly proceeds as follows: (1) at time *t* a motion observation $o_{t}^{\left(1\right),1}$ is sampled from the generative process **A**^(1),1^; (2) posterior beliefs about the motion direction at the current timestep $s_{t}^{\left(1\right),1}$ are updated using a gradient descent on the variational free energy. In addition, we included a second, controllable hidden state factor at Level 1 that we refer to as the abstract “sampling state” of the agent. We include this in order to enable policies at this level, which entail transitions between the two possible values of this control state. These correspond to the choice to either keep sampling the current stimulus or break sampling. These policies are stored as two 2 × 2 transition matrices in **B**^(2),2^, where each transition matrix **B**^(2),2^(*u*)encodes the probability of transitioning to “**Keep-sampling**” or “**Break-sampling**,” given an action *u* and occupancy in one of the two sampling states. Note that these policies only consider actions at the next time step, meaning that the policy-space is identical to the action-space (there is no sequential aspect to the policies). Selecting the first action keeps the Level 1 MDP in the “**Keep-sampling**” state, triggering the generation of another motion observation from the generative process. Engaging the second “**Break-sampling**” policy moves the agent‘s sampling regime into the second state and terminates any further updates at Level 1. At this point the latest posterior beliefs from Level 1 are sent up as observations for Level 2. It is worth noting that implementing “breaking” the MDP at the lower level as an explicit policy departs from the original formulation of deep, temporal active inference. In the formulation developed in Friston et al. (2017d), termination of lower level MDPs occurs once the entropy of the lower-level posterior over the hidden states (only those factors that are linked with the level above) is minimized beyond a fixed value^4^. We chose to treat breaking the first level MDP as an explicit policy in order to formulate behavior in terms of the same principles that drive action selection at the higher level—namely, the expected free energy of policies. In the Simulations section we explore how the dynamic competition between the “**Break-**” and “**Keep-sampling**” policies induces an unexpected distribution of break latencies.

We fixed the maximum temporal horizon of Level 1 (hereafter *T*_1_) to be 20 time steps, such that if the “**Break-sampling**” policy is not engaged before *t* = 20 (implying that “**Keep-sampling**” has been selected the whole time), Level 1 automatically terminates after the 20^th^ time step and the final posterior beliefs are passed up as outcomes for Level 2.

#### 4.2.2. Level 2: Scene Inference and Saccade Selection

After beliefs about the state of the currently-foveated visual region are updated via active inference at Level 1, the resulting posterior belief about motion directions is passed up to Level 2 as a belief about observations. These observations (which can be thought of as the inferred state of the visual stimulus at the foveated area) are used to update the statistics of posterior beliefs over the hidden states operating at Level 2 (specifically, the hidden state factor that encodes the identity of the scene, e.g., **UP-RIGHT**). Hidden states at Level 2 are segregated into two factors, with corresponding posterior beliefs about them updated independently.

**Figure 7:**
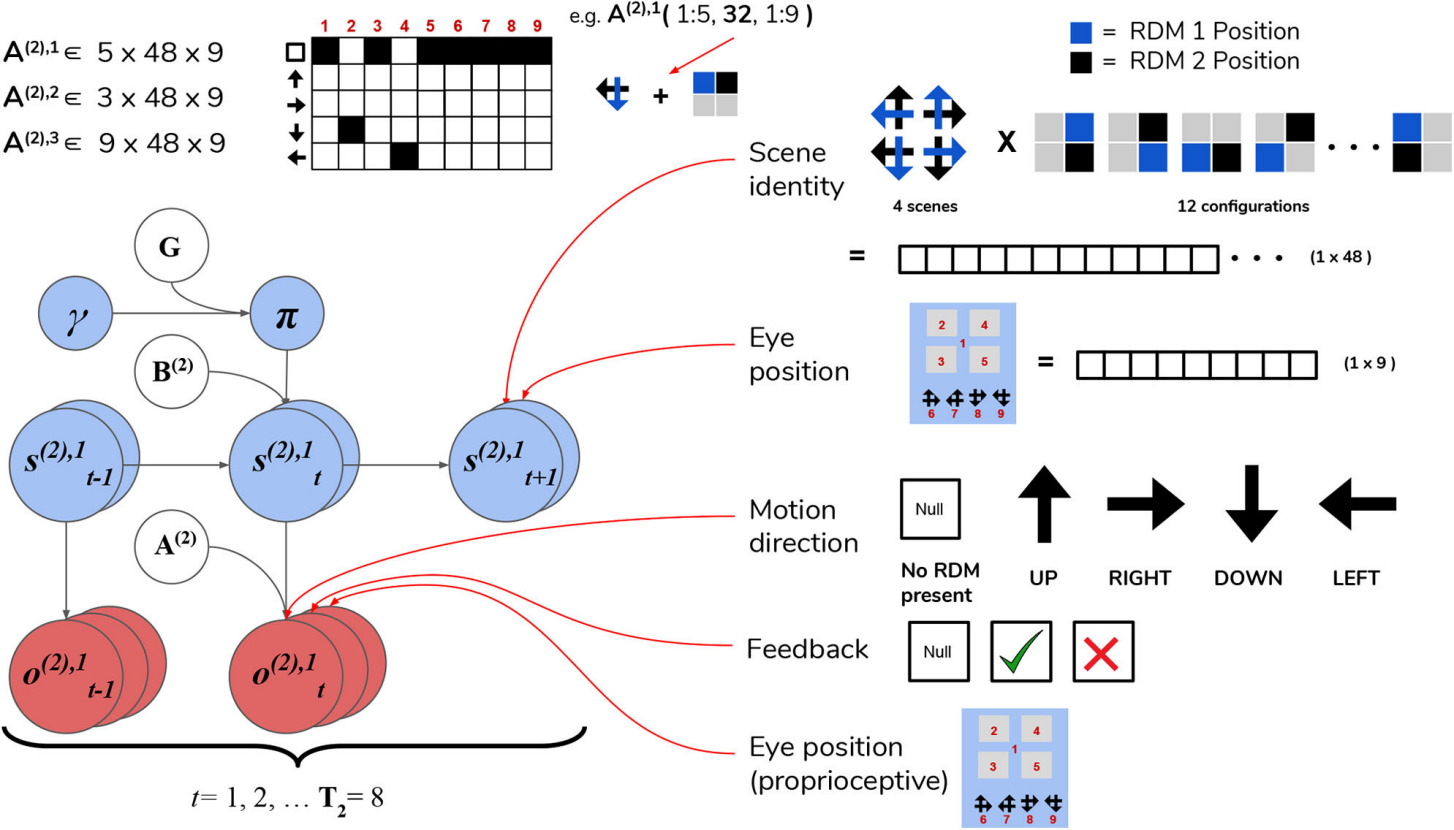
Level 2 MDP. **Level 2** of the hierarchical POMDP for scene construction. Hidden states consist of two factors, one encoding the scene identity and another encoding the eye position (i.e., current state of the oculomotor system). The first hidden state factor **s**^(2),1^ encodes the scene identity of the trial in terms of two unique RDM directions occupy two of the quadrants (four possible scenes as described in the top right panel) and spatial configuration (one of 12 unique ways to place two RDMs in four quadrants). This yields a dimensionality of 48 for this hidden state factor (4 scenes × 12 spatial configurations). The second hidden state factor **s**^(2),2^ encodes the eye position, which is initialized to be in the center of the quadrants (Location 1). The next four values of this factor index the four quadrants (2–5), and the last four are indices for the choice locations (the agent fixates one of these four options to guess the scene identity). As with the sampling state factor at Level 1, the eye position factor **s**^(2),2^ is controllable by the agent through the action-dependent transition matrices **B**^(2),2^. Outcomes at Level 2 are characterized by three modalities: the first modality **o**^(2),1^ indicates the visual stimulus (or lack thereof) at the currently-fixated location. Note that during belief updating, the observations of this modality **o**^(2),1^ are inferred hidden states over motion directions that are passed up after solving the Level 1 MDP (see Figure 6). An example likelihood matrix for this first modality is shown in the upper left, showing the conditional probabilities for visual outcomes when the 1st factor hidden state has the value 32. This corresponds to the scene identity **DOWN-LEFT** under spatial configuration 8 (the RDMs occupy quadrants indexed as Locations 2 and 4). The last two likelihood arrays **A**^(2),2^ and **A**^(2),3^ map to respective observation modalities **o**^(2),2^ and **o**^(2),3^, and are not shown for clarity; the **A**^(2),2^ likelihood encodes the joint probability of particular types of trial feedback (Null, Correct, Incorrect—encoded by **o**^(2),2^) as a function of the current hidden scene and the location of the agent's eyes, while **A**^(2),3^ is an unambiguous proprioceptive mapping that signals to the agent the location of its own eyes via **o**^(2),3^. Note that these two last observation modalities **o**^(2),2^ and **o**^(2),3^ are directly sampled from the environment, and are not passed up as “inferred observations” from Level 1.

The first hidden state factor corresponds to the scene identity. As described in Section 3, there are four possible scenes characterizing a given trial: **UP-RIGHT**, **RIGHT-DOWN**, **DOWN-LEFT**, and **LEFT-UP**. The scene determines the identities of the two RDMs hiding throughout the four quadrants, e.g., when the scene is **UP-RIGHT**, one **UP**wards-moving RDM is found in one of the four quadrants, and a **RIGHT**wards-moving RDM is found in another quadrant. The quadrants that are occupied by RDMs for a given scene is random, meaning that agents have to forage the 2 × 2 array for the RDMs in order to infer the scene. We encode the scene identities as well as their “spatial permutability” (with respect to quadrant-occupancy) by means of a single hidden state factor that exhaustively encodes the unique combinations of scenes and their spatial configurations. This first hidden state factor is thus a 48-dimensional state distribution (4 scenes × 12 possible spatial configurations—see Figure 7 for visual illustration).

The second hidden state factor corresponds to the current spatial position that's being visually fixated—this can be thought of as a hidden state encoding the current configuration of the agent's eyes. This hidden state factor has nine possible states: the first state corresponds to an initial position for the eyes (i.e., a fixation region in the center of the array); the next four states (indices 2–5) correspond to the fixation positions of the four quadrants in the array, and the final four states (6–9) correspond to categorization choices (i.e., a saccade which reports the agent's guess about the scene identity). The states of the first and second hidden state factors jointly determine which observation is sampled at each timestep on Level 2.

Observations at this level comprise three modalities. The first modality encodes the identity of the visual stimulus at the fixated location and is identical in form to the first hidden state factor at Level 1: namely, it can be either the “Null” outcome (when there is no visual stimulus at the fixated location) or one of the four motion directions. The likelihood matrix for the first-modality on Level 2, namely **A**^(2),1^, consists of probabilistic mappings from the scene identity /spatial configuration (encoded by the first hidden state factor) and the current fixation location (the second hidden state factor) to the stimulus identity at the fixated location, e.g., if the scene is **UP-RIGHT** under the configuration where the **UP**wards-moving RDM is in the upper left quadrant and the **RIGHT**wards-moving RDM is in the upper right quadrant and the current fixation location (the second hidden state) is the upper left quadrant, then the likelihood function will determine the first-modality observation at Level 2 to be **UP**. When the agent is fixating either an empty quadrant, the starting fixation location, or one of the response options (locations 6–9), the observation in the first modality is **Null**. The likelihood functions are deterministic and identical in both the generative model and generative process—this imbues the agent with a kind of “prior knowledge” of the (deterministic) mapping between the scenes and their respective visual manifestations in the 2 × 2 grid. The second observation modality is a ternary variable that returns feedback to the agent based on its scene categorization performance—it can assume the values of “No Feedback,” “Correct,” or “Incorrect.” Including this observation modality (and prior beliefs about the relative probability of its different values) allows us to endow agents with the drive to report their guess about the scene, and to do so accurately in order to maximize the chance of receiving correct feedback. The likelihood mapping for this modality **A**^(2),2^ is structured to return a “No Feedback” outcome in this modality when the agent fixates any area besides the response options, and returns “Correct” or “Incorrect” once the agent makes a saccade to one of the response options (locations 6–9)—the particular value it takes depends jointly on the true hidden scene and the scene identity that the agent has guessed. We will further discuss how a drive to respond accurately emerges when we describe the prior beliefs parameterized by the **C** and **D** arrays. The final observation modality at Level 2 is a proprioceptive mapping (similar to “sampling-state” modality at Level 1) that unambiguously signals which location the agent is currently fixating via a 9 × 9 identity matrix **A**^(2),3^.

The transition matrices at Level 2, namely **B**^(2),1^ and **B**^(2),2^, describe the dynamics of the scene identity and of the agent's oculomotor system, respectively. We assume the dynamics that describe the scene identity are both uncontrolled and unchanging, and thus fix **B**^(2),1^ to be an identity matrix that ensures the scene identity/spatial configuration is stable over time. As in earlier formulations (Friston et al., 2012a; Mirza et al., 2016, 2019a) we model saccadic eye movements as transitions between control states in the 2nd hidden state factor. The dynamics describing the eye movement from the current location to a new location is encoded by the transition array **B**^(2),2^ (e.g., if the action taken is 3 then the saccade destination is described by a transition matrix that contains a row of 1s on the third row, mapping from any previous location to location 3).

Inference and action selection at Level 2 proceeds as follows: based on the current hidden state distribution and Level 1's likelihood mapping **A**^(1),1^ (the generative process), observations are sampled from the three modalities. The observation under the first-modality at this level (either “Null” or a motion direction parameterizing an RDM stimulus) is passed down to Level 1 as the initial *true* hidden state. The agent also generates expectations about the first-modality observations via ${\text{A}}^{\left(1\right),1}\cdot Q\left({\text{s}}_{t}\right)$, where **A**^(1),1^ is the generative model's likelihood and *Q*(**s**_*t*_) is the latest posterior density over hidden states (factorized into scene identity and fixation location). This predictive density over (first-modality) outcomes serves as an *empirical prior* for the agent's beliefs about the hidden states in the first factor—motion direction—at Level 1. Belief-updating and policy selection at Level 1 then proceeds via active inference using the empirical priors inherited from Level 2 in addition to its own generative model and process (as described in Section 4.2.1). Once the motion observations and belief updating terminates at Level 1, the final posterior beliefs about the 1^st^ factor hidden states are passed to Level 2 as “inferred” observations of the first modality. The belief updating at Level 2 proceeds as usual, where observations (both those “inferred” from Level 1 and the true observations from the Level 2 generative process: the oculomotor state and reward modality) are integrated using Level 2's generative model to form posterior beliefs about hidden states and policies. The policies at this level, like at the lower level, only consider one step ahead in the future—so each policy consists of one action (a saccade to one of the quadrants or a categorization action), to be taken at the next timestep. An action is sampled from the posterior over policies *a*_*t*_ ~ *Q*(π), which changes hidden states in the next time step to generate a new observation, thus closing the action-perception cycle. In this spatiotemporally “deep” version of scene construction, we see how a temporally-extended process of active inference at the lower level (capped at **T**_1_ = 20 time steps in our case) can be nested within a single time step of a higher-level process, endowing such generative models with a flexible, modular form of temporal depth. Also note the asymmetry in informational scheduling across layers, with posterior beliefs about those hidden states linked with the higher level being passed *up* as evidence for outcomes at the higher level, with observations at the higher level being passed *down* as empirical priors over hidden states at the lower level.

#### 4.2.3. Priors

**Figure 8:**
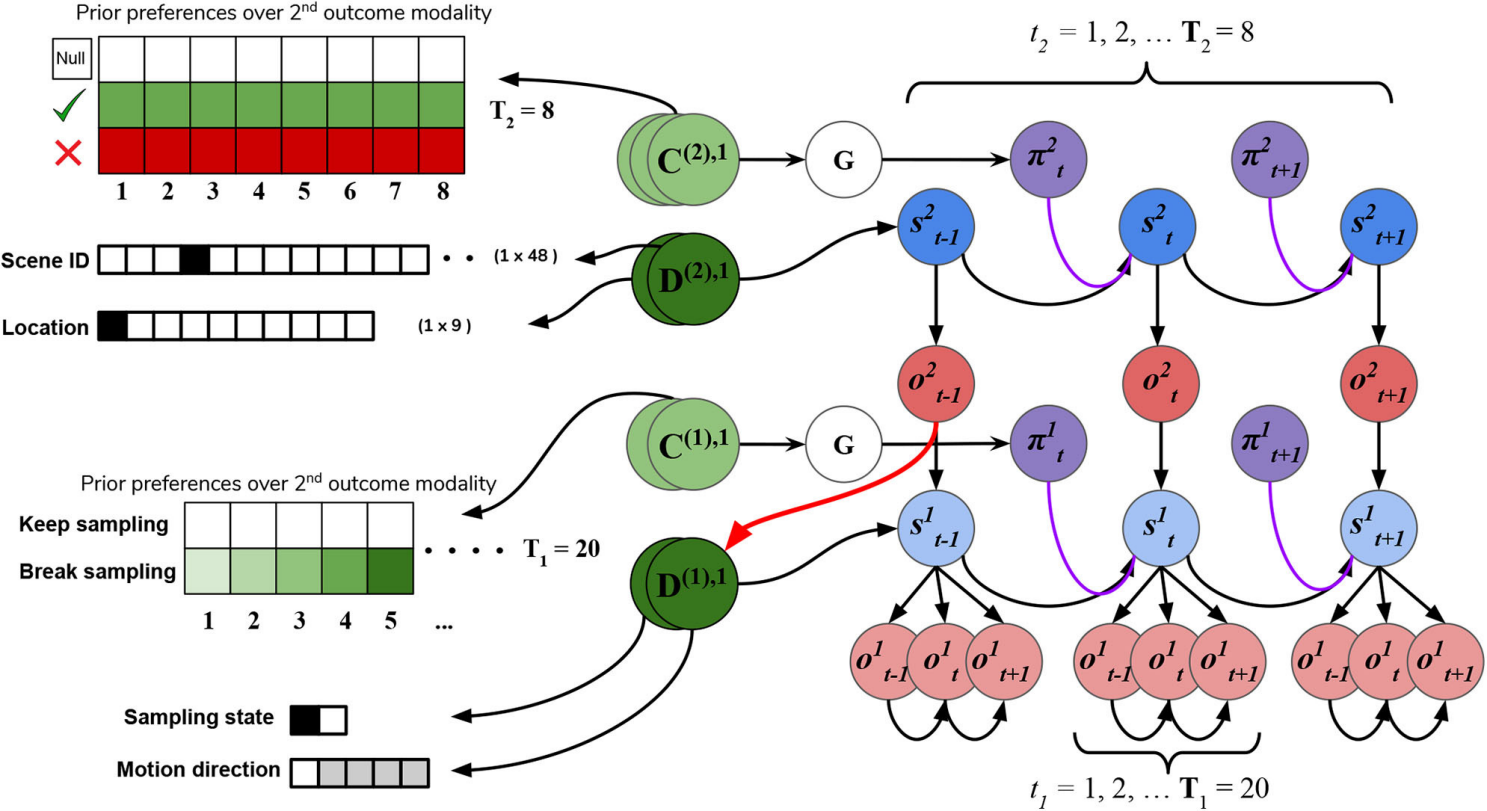
**C**'s and **D**'s. Prior beliefs over observations and hidden states for both hierarchical levels. Note that superscripts here index the hierarchical level, and separate modalities/factors for the **C** and **D** matrices are indicated by stacked circles. At the highest level (Level 2), prior beliefs about second-modality outcomes (**C**^(2),2^) encode the agent's beliefs about receiving correct and avoiding incorrect feedback. Prior beliefs over the other outcome modalities (**C**^(2),1^ and **C**^(2),3^) are all trivially zero. These beliefs are stationary over time and affect saccade selection at Level 2 via the expected free energy of policies **G**. Prior beliefs about hidden states **D**^(2)^ at this level encode the agent's initial beliefs about the scene identity and the location of their eyes. This prior over hidden states can be manipulated to put the agent's beliefs about the world at odds with the actual hidden state of the world. At Level 1, the agent's preferences about being in the “**Break-sampling**” state increases over time and is encoded in the preferences about second modality outcomes (**C**^(1),2^), which corresponds to the agents umambiguous perception of its own sampling state. Finally, the prior beliefs about initial states at Level 1 (**D**^1^) correspond to the motion direction hidden state (the RDM identity) as well as which sampling-state the agent is in. Crucially, the first factor of these prior beliefs **D**^(1),1^ is initialized as the “expected observations” from Level 2: the expected motion direction (first modality). These expected observations are generated by passing the variational beliefs about the scene at Level 2 through the modality-specific likelihood mapping: *Q*(*o*^(2),1^|*s*^(2),1^) = *P*(*o*^(2),1^|*s*^(2),1^)*Q*(*s*^(2),1^). The prior over hidden states at Level 1 is thus called an *empirical prior* as it is inherited from Level 2. The red arrow indicates the relationship between the expected observation from Level 2 and the empirical prior over (first-factor) hidden states at Level 1.

In addition to the likelihood **A** and **B** arrays that prescribe the probabilistic relationships between variables at each level, the generative model is also equipped with prior beliefs over observations and hidden states that are respectively encoded in the so-called **C** and **D** arrays. See Figure 8 for schematic analogies for these arrays and their elements for the two hierarchical levels.

The **C** array contains what are often called the agent's “preferences” *P*(*o*) and encodes the agent‘s prior beliefs about observations (an unconditional probability distribution). Rather than an explicit component of the generative model, the prior over outcomes is absorbed into the prior over policies *P*(π), which is described in Section 2.2. Policies that are more likely to yield observations that are deemed probable under the prior (expressed in terms of agent's preferences *P*(*o*)) will have less expected free energy and thus be more likely to be chosen. *Instrumental value* or expected utility measures the degree to which the observations expected under a policy correlate with prior beliefs about those observations. For categorical distributions, evaluating instrumental value amounts to taking the dot product of the (policy-conditioned) posterior predictive density over observations *Q*(*o*_τ_|π) with the log probability density over outcomes log*P*(*o*_τ_). This reinterpretation of preferences as prior beliefs about observations allows us to discard the classical notion of a “utility function” as postulated in fields like reward neuroscience and economics, instead explaining both epistemic and instrumental behavior using the common currency of log-probabilities and surprise. In order to motivate agents to categorize the scene, we embed a self-expectation of accuracy into the **C** array of Level 2; this manifests as a high prior expectation of receiving “Correct” feedback (a relative log probability of +2 nats) and an expectation that receiving “Incorrect” feedback is unlikely (relative log probability of −4 nats). The remaining outcomes of the other modalities at Level 2 have equal log-probability in the agent's prior preferences, thus contributing identically (and uninformatively) to instrumental value. At Level 1 we encoded a form of “urgency” using the **C** matrix; we encoded the prior belief that the probability of observing the “**Break-sampling**” state (via the umambiguous mapping *A*^(1),2^) increases over time. This necessitates that the complementary probability of remaining in the “**Keep-sampling**” state decreases over time. Equipping the Level 1 MDP with such preferences generates a tension between the epistemic drive to resolve uncertainty about the hidden state of the currently-fixated stimulus and the ever-strengthening prior preference to terminate sampling at Level 1. In the simulation results to follow, we explore this tension more explicitly and report an interesting yet unexpected relationship between sensory uncertainty and fixational dwell time, based on the dynamics of various contributions to expected free energy.

Finally, the **D** array encodes the agent's initial (prior) beliefs over hidden states in the environment. By changing prior beliefs about the initial states, we can manipulate an agent's beliefs about the environment independently of the true hidden states characterizing that environment. In the Section 5.2 below we describe the way we parameterize the first hidden state factor of the Level 2 **D** matrix to manipulate prior beliefs about the scene. The second hidden state factor at Level 2 (encoding the saccade location) is always initialized to start at Location 1 (the generic “starting” location). At Level 1, the first-factor of the **D** matrix (encoding the true motion direction of an RDM) is initialized to the posterior expectations from Level 2, i.e., $Q\left(o^{\left(1\right),1}|{\text{s}}_{t}\right)={\text{A}}^{\left(1\right),1}Q\left({\text{s}}_{t}\right)$. The second-factor belief about hidden states (encoding the sampling state) is initialized to the “**Keep-sampling**” state.

In the following sections, we present hierarchical active inference simulations of scene construction, in which we manipulate the uncertainty associated with beliefs at different levels of the generative model to see how uncertainty differentially affects inference across levels in uncertain environments.

## 5. Simulations

Having introduced the hierarchical generative model for our RDM-based scene construction task, we will now explore behavior and belief-formation in the context of hierarchical active inference. In the following sections we study different aspects of the generative model through quantitative simulations. We relate parameters of the generative model to both “behavioral” read-outs (such as sampling time, categorization latency and accuracy) as well as the agents' internal dynamics (such as the evolution of posterior beliefs, the contribution of different kinds of value to policies, etc.). We then discuss the implications of our model for studies of hierarchical inference in noisy, compositionally-structured environments.

### 5.1. Manipulating Sensory Precision

**Figure 9:**
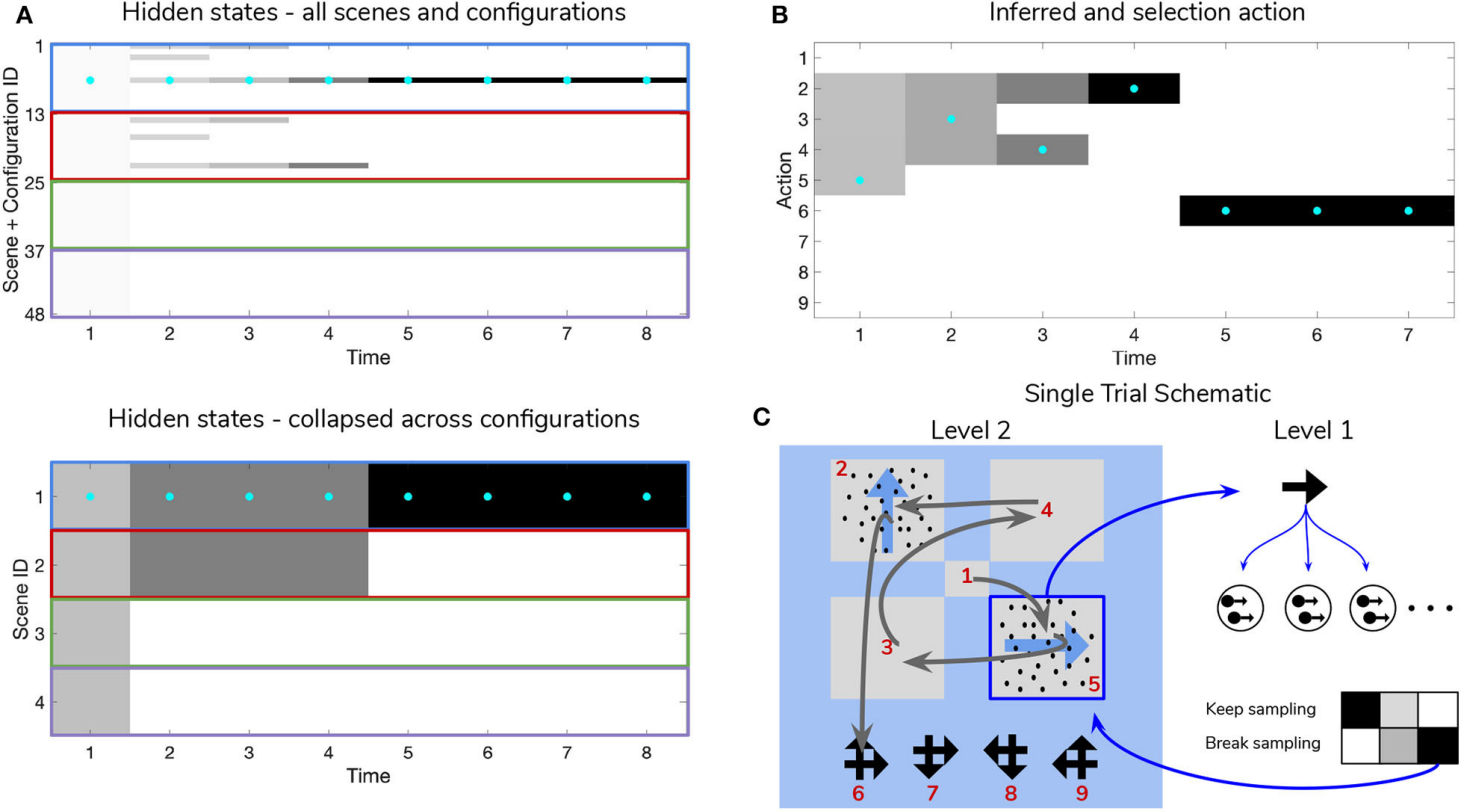
Simulated trial of scene construction under high sensory precision. **(A)** The evolution of posterior beliefs about scene identity—the first factor of hidden states at Level 2—as a deep active inference agent explores the visual array. In this case, sensory precision at Level 1 is high, meaning that posterior beliefs about the motion direction of each RDM-containing quadrant are resolved easily, resulting in fast and accurate scene categorization. Cells are gray-scale colored according to the probability of the belief for that hidden state and time index (darker colors correspond to higher probabilities). Cyan dots indicates the true hidden state at each time step. The top row of **(A)** shows evolving beliefs about the fully-enumerated scene identity (48 possibilities), with every 12 configurations highlighted with a differently-colored bounding box, correspond to beliefs about each type of scene (i.e., **UP-RIGHT**, **RIGHT-DOWN**, **DOWN-LEFT**, **LEFT-UP**). The bottom panel shows the collapsed beliefs over the four scenes, computed by summing the hidden state beliefs across the 12 spatial configurations. **(B)** Evolution of posterior beliefs about actions (fixation starting location not shown), culminating in the categorization decision (here, the scene was categorized as **UP-RIGHT**, corresponding to a saccade to location 6. **(C)** Visual representation of the agent's behavior for this trial. Saccades are depicted as curved gray lines connecting one saccade endpoint to the next. Fixation locations (corresponding to 2nd factor hidden state indices) are shown as red numbers. The Level 1 active inference process occurring within a single fixation is schematically represented on the right side, with individual motion samples shown as issued from the true motion direction via the low level likelihood **A**^(1),1^. The agent observes the true RDM at Level 1 and updates its posterior beliefs about this hidden state. As uncertainty about the RDM direction is resolved, the “**Break-sampling**” action becomes more attractive (since epistemic value contributes increasingly less to the expected free energy of policies). In this case, the sampling process at Level 1 is terminated after only three timesteps, since the precision of the likelihood mapping is high (*p* = 5.0) which relates to the speed at which uncertainty is resolved about the RDM motion direction—see the text for more details.

**Figure 10:**
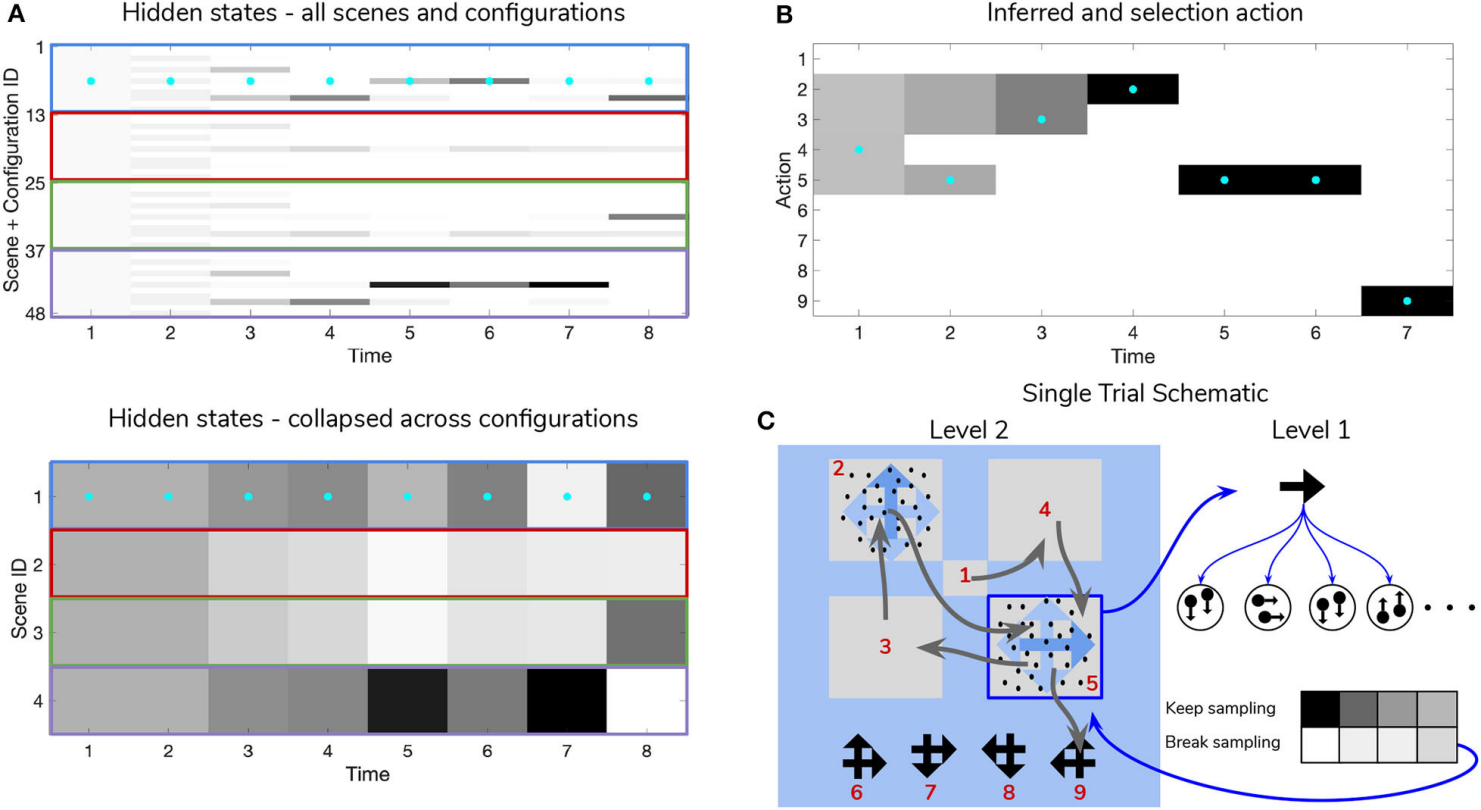
Simulated trial of scene construction with low sensory precision. Same as in Figure 9, except in this trial the precision of the mapping between RDM motion directions and samples thereof is lower, *p* = 0.5. This leads to an incorrect sequence of inferences, where the agent ends up believing that the scene identity is **LEFT-UP** and guessing incorrectly. Note that after this choice is made and incorrect feedback is given, the agent updates their posterior in terms of the “next best” guess, which is from the agent's perspective either **UP-RIGHT** or **DOWN-LEFT** (see the posterior at Time step 8 of **(A)**). **(C)** Shows that the relative imprecision of the Level 1 likelihood results in a sequence of stochastic motion observations that frequently diverge from the true motion direction (in this case, the true motion direction is **RIGHT** in the lower right quadrant (Location 5)). Level 1 belief-updating gives rise to an imprecise posterior belief over motion directions that are passed up as inferred outcomes to Level 2, leading to false beliefs about the scene identity. Note the “ambivalent,” quadrant-revisiting behavior, wherein the agent repeatedly visits the lower-right quadrant to resolve uncertainty about the RDM stimulus at that quadrant.

Figures 9, 10 show examples of deep active inference agents performing the scene construction task under two levels of motion coherence (high and low, respectively for Figures 9, 10), which is equivalent to the reliability of motion observations at Level 1. In particular, we operationalize this uncertainty via an inverse temperature *p* that parameterizes a softmax transformation on the columns of the Level 1 likelihood mapping to RDM observations **A**^(1),1^. Each each column of **A**^(1),1^ is initialized as a “one-hot” vector that contains a probability of 1 at the motion observation index corresponding to the true motion direction, and 0s elsewhere. As *p* decreases, **A** deviates further from the identity matrix and Level 1 motion observations become more degenerate with respect to the hidden state (motion direction) underlying them. Note that this parameterization of motion incoherence only pertains to the last four rows/columns of **A**^(1),1^, as the first row/column of the likelihood (**A**^(1),1^(1, 1)) corresponds to observations about the “Null” hidden state, which is always observed unambiguously when it is present. In other words, locations that do not contain RDM stimuli are always perceived as “Null” in the first modality with certainty.

Figure 9 is a simulated trial of scene construction with sensory uncertainty at the lower level set to *p* = 5.0. This manifests as a stream of motion observations at the lower level that reflect the true motion state ~98% of the time, i.e., highly-coherent motion. As the agent visually interrogates the 2 × 2 visual array (the 2^nd^ to 5^th^ rows of Panel **B**), posterior beliefs about the hidden scene identity (Panel **A**) converge on the true hidden scene. After the first RDM in the lower right quadrant is seen (and its state resolved with high certainty), the agent's Level 2 posterior starts to only assign non-zero probability to scenes that include the **RIGHT**wards-moving motion stimulus. Once the second, **UP**wards-moving RDM stimulus is perceived in the upper left, the posterior converges upon the correct scene (in this case, indexed as state 7, one of the 12 configurations of **UP-RIGHT**). Once uncertainty about the hidden scene is resolved, **G** becomes dominated by instrumental value, or the dot-product of counterfactual observations with prior preferences. Expecting to receive correct feedback, the agent saccades to location 6 (which corresponds to the scene identity **UP-RIGHT**) and receives a “Correct” outcome in the second-modality of Level 2 observations. The agent thus categorizes the scene and remains there for the remainder of the trial to exploit the expected instrumental value of receiving “Correct” feedback (for the discussion about how behavior changes with respect to prior belief and sensory precision manipulations, we only consider behavior up until the time step of the first categorization decision).

**Figure 11:**
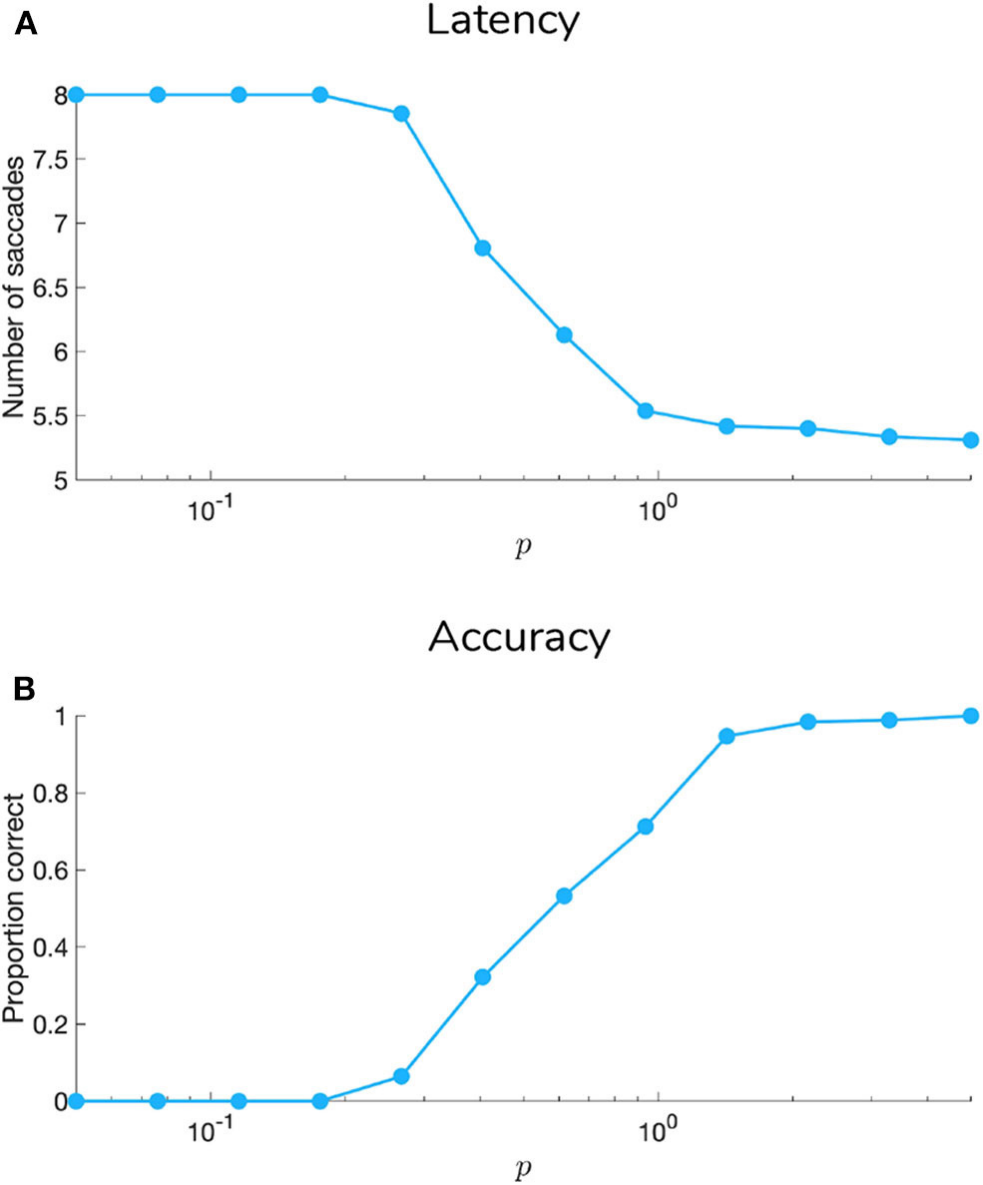
Effect of sensory precision on scene construction performance. Average categorization latency **(A)** and accuracy **(B)** as a function of sensory precision *p* which controls the entropy of the (Level 1) likelihood mapping from motion direction to motion observation. We simulated 185 trials of scene construction under hierarchical active inference for each level of *p* (12 levels total), with scene identities and configurations randomly initialized for each trial. Sensory precision is shown on a logarithmic scale.

Figure 10 shows a trial when the RDMs are incoherent (*p* = 0.5, meaning the Level 1 likelihood yields motion observations that reflect the true motion state ~35% of the time). In this case, the agent fails to categorize the scene correctly due to the inability to form accurate beliefs about the identity of RDMs at Level 1—this uncertainty carries forward to lead posterior beliefs at Level 2 astray. Interestingly, the agent still forms relatively confident posterior beliefs about the scene (see the posterior at Timestep 7 of Figure 11A), but they are inaccurate since they are based on inaccurate posterior beliefs inherited from Level 1. This is because even though the low-level belief is built from noisy observations, posterior probability ends up still “focusing” on a particular dot direction based on the particular sequence of observations that is sampled; this is then integrated with empirical priors and subsequent observations to narrow the possible space of beliefs about the scene. The posterior uncertainty also manifests as the time spent foraging in quadrants before making categorization (nearly double the time spent by the agent in Figure 9). The cause of this increase in foraging time is 2-fold. First of all, since uncertainty about the scene identity is high, the epistemic value of policies that entail fixations to RDM-containing quadrants remains elevated, even after all the quadrants have been visited. This is because uncertainty about hidden states is unlikely to be resolved after a single saccade to a quadrant with an incoherent RDM, meaning that the epistemic value of repeated visits to such quadrants decreases slowly with repeated foraging. Secondly, since Level 2 posterior beliefs about the scene identity are uncertain and are distributed among different states, the instrumental value of categorization actions remains low—remember that instrumental value depends not only on the instrumental value of receiving “Correct” feedback, but also on the agent's expectation about the *probability* of receiving this feedback upon making an action, relative to the probability of receiving “Incorrect” feedback. The relative values of the prior preferences for being “Correct” vs. “Incorrect” thus tune the risk-averseness of the agent, and manifest as a dynamic balance between epistemic and instrumental value. See Mirza et al. (2019b) for a quantitative exploration of these prior preferences and their effect on active inference.

We quantified the relationship between sensory precision and scene construction performance by simulating scene construction trials under different sensory precisions *p* (see Figure 11). The two measures shown are: (1) *categorization latency* (Figure 11A), defined as the number of time steps elapsed before a saccade to one of the choice locations is initiated; and (2) *categorization accuracy* (Figure 11B), defined as percentage of trials when the agent's first categorization resulted in “Correct” feedback. In agreement with intuition, for low values of *p* agents take more time to categorize the scene and categorize less accurately. As sensory precision increases, agents require monotonically less time to forage the array before categorizing, and this categorization also becomes more accurate. In the next section, we explore the relationship between sensory precision and performance when the agent entertains prior beliefs of varying strength about the probability of a certain scene.

### 5.2. Manipulating Prior Beliefs

**Figure 12:**
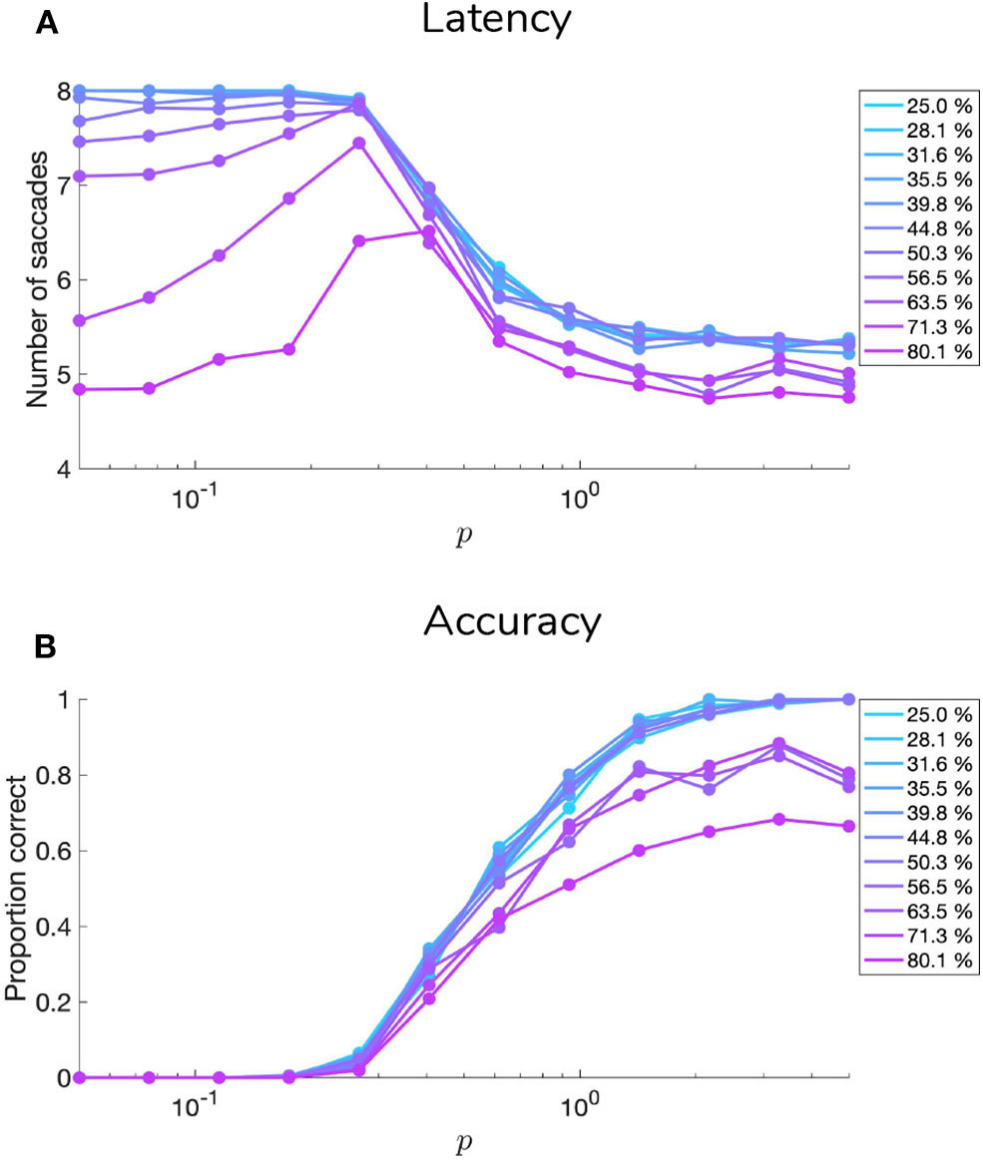
Effect of sensory precision on scene construction performance for different prior belief strengths. Same as in Figure 11 but for different strengths of initial prior beliefs (legend on right). Prior belief strengths are defined as the probability density of the prior beliefs about hidden states (1st hidden state factor of Level 2—**D**^(2),1^) concentrated upon one of the four possible scenes. This elevated probability is uniformly spread among the 12 hidden states corresponding to the different quadrant-configurations of that scene, such that the agent has no prior expectation about a particular arrangement of the scene, but rather about that scene type in general. Here, we only show the results for agents with “incorrect” prior beliefs—namely, when the scene that the agent believes to be at play is different from the scene actually characterizing the trial.

For the simulations discussed in the previous section, agents always start scene construction trials with “flat” prior beliefs about the scene identity. This means that the first factor of the prior beliefs about hidden states at Level 2 **D**^(2),1^ was initialized as a uniform distribution. We can manipulate the agent's initial expectations about the scenes and their spatial arrangements by arbitrarily sculpting **D**^(2),1^ to have high or low probabilities over any state or set of states. Although many manipulations of the Level 2 prior over hidden states are possible, here we introduce a simple prior belief manipulation by uniformly elevating the prior probability of all spatial configurations (12 total) of a single type of scene. For example, to furnish an agent with the belief that there's a 50% chance of any given trial being a **RIGHT-DOWN** scene, we simply boost the probabilities associated with hidden states 13–25 (the 12 spatial configurations of the **RIGHT-DOWN** scene) relative to the other hidden scenes, so that the total integrated probability of hidden states 13–25 is 0.5. This implies that the other hidden scenes each now have $\frac{\left(1-0.5\right)}{3}\approx 0.1667$ probability, once respectively integrated over their 12 configuration states. Figure 12 shows the effect of parametrically varying the strengths of prior beliefs on the same behavioral measures shown in Figure 11. Similar to Figures 11, 12 demonstrates a monotonic increase in accuracy with increasing sensory precision, regardless of how much the agent initially expects a particular scene type. This means that strong but incorrect prior beliefs (over initial states) can still be “overcome” with reliable enough sensory data. However, agents with stronger priors are less sensitive to the increase in sensory precision than their “flat-priored” counterparts, as can be seen by the lower accuracy level of the most purple-colored lines in Figure 12. Note that the averages shown are only for agents with “incorrect” prior beliefs; namely, the prior over hidden states in the generative model for each trial was always initialized to be a different scene type than the true scene. This has the effect of setting the minimum accuracy for the “strongest-priored” agents (who typically categorize the scene identity at the first time step) at 0% rather than 25% (chance performance). These results are consistent with the fundamental relationship between the likelihood term and prior probability in Bayes' theorem (see Equation 1): the posterior over hidden states is calculated as the product of the likelihood and the prior. Increasing the precision of one of these two will “shift” the posterior distribution in the respective direction of the more precise distribution. This manifests as a parametric “de-sensitizing” of posterior beliefs to sensory evidence as priors become stronger. This balance between sensory and prior precision is exactly manifested in the prior-dependent sensitivity of the accuracy curves in Figure 12B.

The interaction between sensory and prior precision is not as straightforward when it comes to categorization latency. Figure 12A shows that when the sensory precision *p* is high enough, most of the variance in latency introduced by prior beliefs vanishes, since observations alone can be relied on to ensure fast inference about the scene. For low values of *p*, however, latency is highly-sensitive to prior belief strength. Under weak prior beliefs and low *p*, the agent displays ambivalence—beliefs about RDM direction at Level 1 are not precise enough to enable scene inference, causing the agent to choose the policies that have (albeit) small epistemic value while avoiding the risk of categorizing incorrectly. This causes the agent to saccade among RDM-containing quadrants. Agents with stronger prior beliefs, however, do not rely on observations to determine posterior beliefs because their prior beliefs about the scene already lend high instrumental value to categorization actions. This corresponds to trials when the agent categorizes the scene immediately (for the strongest prior beliefs, this occurs even before inspecting any quadrants) and relying minimally on sensory evidence. This faster latency comes at the cost of accuracy, however, as evident from the lower average accuracy of strongly-priored agents displayed in Figure 12B.

**Figure 13:**
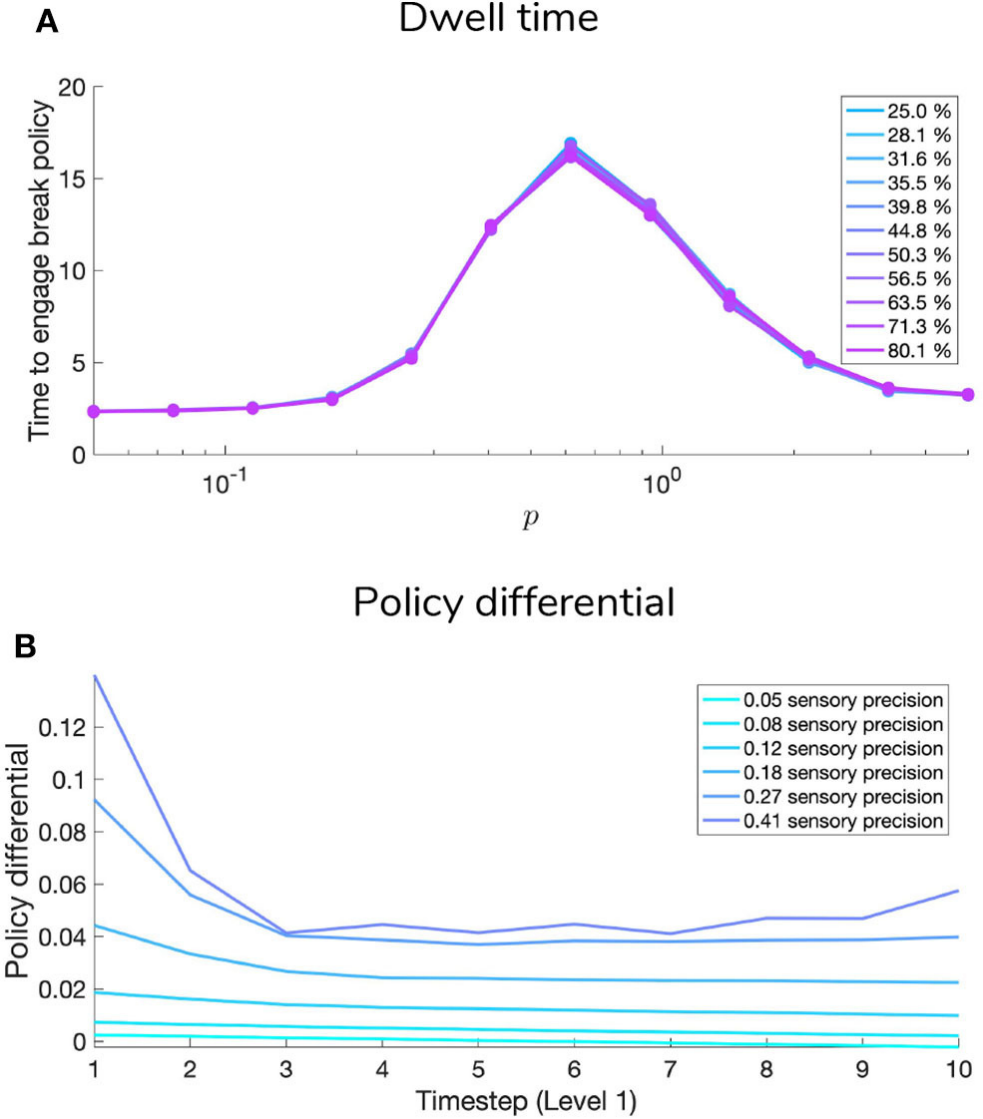
Effect of sensory precision on quadrant dwell time. **(A)** Shows the effect of increasing sensory precision at Level 1 on the time it takes to switch to “**Break-sampling**” policy. Here, 250 trials were simulated for each combination of sensory precision and prior belief strength, with priors over hidden states at Level 2 randomly initialized to have high probability about 1 of the 4 scene types. Break-times were analyzed only for the first saccade (at Level 2) of each trial. **(B)** Shows the effect of sensory precision on evolution of the relative posterior probabilities of the “**Keep-sampling**” vs. the “**Break-sampling**” policies (*Policy Differential* = *P*_Keep-sampling_−*P*_Break-sampling_). We only show these posterior policy differentials for the first 10 time steps of sampling at Level 1 due to insufficient numbers of saccades that lasted more than 10 time steps at the highest/lowest sensory precisions (see **A**). Averages are calculated across different prior belief strengths, based on the lack of an effect, as is apparent in **(A)**. The policy differential defined in this way is always positive because as soon as the probability of “**Break-sampling**” exceeds that of “**Keep-sampling**” (i.e., *Policy Differential* < 0), the “**Break-sampling**” policy will be engaged with near certainty. This is due the high precision over policies at the lower level (here, γ = 512), which essentially ensures that the policy with higher probability will always be selected.

Now we explore the effects of sensory and prior precision on belief-updating and policy selection at the lower level, during a single quadrant fixation. Figure 13A shows the effect of increasing *p* on the break-time (or to analogize it more directly to eye movements: the fixational “dwell time”) at Level 1. We observe a non-trivial, inverted-U relationship between the logarithm of *p* (our analog of motion coherence) and the time it takes for agents to break the sampling at Level 1. For the lowest (most incoherent) values of the likelihood precision *p*, the agents dwell for as little time as they do as for the highest precisions. Understanding this paradoxical effect requires a more nuanced understanding of epistemic value. In general, increasing the precision of the likelihood mapping increases the amount of uncertainty that observations can resolve about hidden states, thus lending high epistemic value to policies that disclose such observations (Parr and Friston, 2017). An elevated epistemic value predicts an increase in dwell time (i.e., via an increase in the epistemic value for the “**Keep-sampling**” policy at Level 1) for increasing sensory precision. However, an increased precision of the Level 1 likelihood also implies that posterior uncertainty is resolved at a faster rate (due to high mutual information between observations and hidden states), which suppresses epistemic value over time. The rate at which epistemic value drops off thus increases in the presence of informative observations, since the posterior converges to a tight probability distribution relatively quickly. On the other hand, at very low likelihood precisions, the low information content of observations in addition to the linearly-increasing cost of sampling (encoded in the Level 1 preferences **C**^(1),2^) renders the sampling of motion observations relatively useless for agents, and it “pays” to just break sampling early. This results in the pattern of break-times that we observe in Figure 13A.

It is worth mentioning the barely noticeable effect of prior beliefs (Figure 13A) about the scene identity on break times at Level 1. Although prior beliefs about the scene at Level 1 manifest as empirical priors over hidden states (motion directions) at Level 2, it seems that the likelihood matrix plays a much larger role in determining break times than the initial beliefs. This means that even when the agent initially assigns relatively more probability to particular RDM directions (conditional on beliefs about scenes at Level 2), this initial belief can quickly be revised in light of incoming evidence (namely, observations at Level 1, inverted through the likelihood mapping to produce a marginal posterior over hidden states). This also speaks to the segregation of belief-updating between hierarchical levels; although beliefs about hidden states and observations are passed up and down the hierarchy, belief-updating occurs only with respect to the variational free energy of a particular layer's generative model, thus insulating variational updating to operate at distinct spatiotemporal scales. This results in the conditional independence of decision-making across hierarchical levels, and clarifies the dissociable influence of prior about scenes on Level 1 vs. Level 2. For example, even on trials when an agent has strong prior beliefs about the scene and thus takes fewer saccades to categorize it, differences in lower-level “dwell time” are still largely determined by the sensory precision *p* of the likelihood mapping and the preference to enter the “**Break-sampling**” state, encoded as an increasing probability to observe oneself occupying this state (in **C**^(1),2^).

The curves in Figure 13B clarify the rate at which epistemic value decreases for high sensory precisions. The “policy differential” measures the difference between the posterior probability of the “**Keep-sampling**” vs. “**Break-sampling**” policies at Level 1: *P*_Keep-sampling_−*P*_Break-sampling_. At the lowest sensory precisions, there is barely any epistemic value to pursuing the “**Keep-sampling**” policy, allowing the break policy to increasingly dominate action-selection over time. For higher sensory precisions, the “**Keep-sampling**” policy starts with >10% more probability than the “**Break-sampling**” policy since the epistemic value of sampling observations is high, but quickly loses its advantage as posterior uncertainty is resolved. At this point the probability of breaking becomes more probable, since posterior beliefs about the RDM are fairly resolved and the instrumental of breaking is only getting higher with time.

## 6. Discussion

In the current work, we presented a hierarchical partially-observed Markov Decision Process model of scene construction, where scenes are defined as arbitrary constellations of random dot motion (RDM) stimuli. Inspired by an earlier model of scene construction (Mirza et al., 2016, 2018) and a deep temporal formulation of active inference (Friston et al., 2017d), we cast this scene construction task as approximate Bayesian inference occurring across two hierarchical levels of inference. One level involves optimizing beliefs about the instantaneous contents of agent-initiated visual fixations; the second level involves integrating the contents of different fixated locations to form beliefs about a higher-level concept like a scene. Through simulations we showed how this deep, temporal model formulation can be used to provide an active inference account of behavior in such compositional inference tasks. Deep active inference agents performing scene construction exhibit the Bayesian hallmarks of a dynamic trade-off between sensory and prior precision when it comes to scene inference and saccade selection. The hierarchical segregation of inference between saccadic and fixational levels gives rise to unexpected effects of sensory uncertainty at the level of single fixations, where we observe an inverted-U relationship between motion coherence and fixational dwell time. This non-linear relation can be explained by appealing to the evolution of epistemic value over time, under the assumption that the agent entertains beliefs about the precision of the environmental process generating visual sensations, while simultaneously optimizing the sufficient statistics of beliefs about the currently-fixated stimulus. The fact that the precision of the likelihood mapping increases the epistemic value of policies that furnish observations sampled from the generative process, while simultaneously increasing the rate at which posterior uncertainty is reduced, explains the non-monotonic influence of sensory precision on Level 1 decision latency.

These results contrast with the predictions of classic evidence accumulation models like the drift-diffusion model or DDM (Ratcliff, 1978; Palmer et al., 2005; Ratcliff and McKoon, 2008). In the drift-diffusion model, reaction times are modeled as proportional to the latency it takes for a time-varying decision variable (or **DV**) to reach one of two fixed decision boundaries **Z** and **−Z** that respectively correspond to two hypotheses (e.g., the equivalent of sufficiently-strong posterior beliefs in one of two hidden states). At each time step, increments to the **DV** are calculated as the log of the ratio between the evidence for each hypothesis conditioned on observations. In discrete-time environments this update-rule for **DV** is equivalent to the Sequential Probability Ratio Test formulated by Wald and Wolfowitz (1948). For time-independent decision boundaries and a fixed initial value of the **DV**, a drift-diffusion process yields a monotonic decreasing relationship between motion incoherence and decision latency (Bogacz et al., 2006; Ratcliff and McKoon, 2008), where motion coherence factors into the DDM as the drift rate of the **DV**—this is analogous to the *sensitivity* of the **DV** to incoming sensory evidence. In the current active inference model, we have binarized policies at Level 1 in part to invite comparison between our model and DDM models (which in their classical form handle binary hypotheses). Rather than modeling actions as discrete perceptual decisions about the most likely hidden state underlying observations (since in the current context, we have a 4-dimensional RDM state space), we instead model the decision as selecting between one of two “sampling” policies, whose probabilities change over time due to the dynamics of the expected free energy. This evolving action-probability weighs epistemic drives to resolve uncertainty against prior preferences that encode an increasing “urgency” to break sampling. This parameterization of decision-making permits a flexible (and in this case, somewhat unexpected) relationship between sensory uncertainty and decision latency (see Figure 13). We thus provide a novel, principled prediction for the relationship between sensory uncertainty and reaction time at different levels of inference in perceptual decision-making tasks.

A discussion of the relationship between the current model and previous hierarchical POMDP schemes is also warranted. The model most closely related to the current work is the “deep temporal model” of active reading, proposed by Friston et al. (2017d); the inference schemes are identical, with the critical difference being the way in which updating is terminated at the lower level. In Friston et al. (2017d), policies at the lower level are driven purely by epistemic value and terminate as a result of posterior uncertainty being reduced beyond a certain pre-determined level. In contrast, the current model introduces an additional “Break-policy” (and corresponding observations of a “Sampling-state”) at the lower level, whose selection is used to terminate the Level 1 POMDP. This also allows us to motivate decision-making at the lower level MDP using individual costs or goals, as encoded via the “sampling cost” in the lower level prior over observations *P*(*o*), explicitly pitting the epistemic drive to resolve uncertainty about the currently-fixated RDM stimulus against the increasing cost of continuing to fixate. Qualitatively, we found that this leads to a smoother relationship between sensory uncertainty (inverse precision of the Level 1 **A** matrix) and the latencies to engage the break policy (“reaction times”), allowing easier comparison of the current model to other evidence accumulation schemes (e.g., drift-diffusion models).

Insight from the robotics and probabilistic planning literature could also be integrated with the current work to extend deep active inference in its scope and flexibility. For instance, the framework of “planning to see” proposed in Sridharan et al. (2010) can be used to drive selective visual processing of goal-relevant features in the sensorium, an important context-sensitive aspect of visual processing (selective and feature-based attention) that is lacking in the current formulation. Mirza et al. (2019a) introduces an active inference model of selective attention in a visual foraging task; the approach proposed therein might be combined with a hierarchical scheme to generate a fully hierarchical model with goal-driven attention operating at multiple levels.

The hierarchical active inference scheme could also be extended to dynamic environments, where the scene itself changes, either due to intrinsic stochasticity or as a function of the agent's (or other agents') actions. This could simply be changed by encoding appropriate self-initiated state-changes into the transition model (the “B” matrices) or by introducing intrinsic, non-agent-controlled dynamics into the generative process. Ongoing work in the robotics and planning literature has highlighted the challenges of dynamic, structured environments and proposed various schemes to both plan actions and form probabilistic beliefs in such tasks (Ognibene and Demiris, 2013; Ognibene and Baldassare, 2014). Future research might find ways to meaningfully integrate existing approaches from the hierarchical planning and POMDP literature with deep active inference models, such as the one proposed here.

In future investigations, we plan to estimate the parameters of hierarchical active inference models from experimental data of human participants performing a scene construction task, where the identities of visual stimuli are uncertain (the equivalent of manipulating the sensory likelihood at Level 1 of the hierarchy). Data-driven inversion of a deep scene construction model can then be used to explain inter-subject variability in aspects of hierarchical inference behavior as different parameterizations of subject-specific generative models.

## Data Availability Statement

The data used in this study are the results of numerical simulations, and as such, we do not provide datasets. The software used to simulate the data and generate associated figures are based on visual foraging and scene construction demos included in SPM v12.0, and can be freely downloaded from https://www.fil.ion.ucl.ac.uk/spm/ as part of the DEM toolbox. The particular versions of these scripts used to implement the deep hierarchical version are available upon request from the authors.

## Author Contributions

RH and AP conceived the original idea for the project. RH and MM conceived the hierarchical active inference model. RH, AP, and IK designed the scene construction task using random dot motion. MM, TP, and KF gave the critical insight into formulation of the model. RH conducted the simulations and analyzed the results. All authors contributed to the writing of the manuscript.

## Conflict of Interest

The authors declare that the research was conducted in the absence of any commercial or financial relationships that could be construed as a potential conflict of interest.

## Footnotes

^1^From now on we assume the use of discrete probability distributions for convenience and compatibility with the sort of generative models relevant to the current work.

^2^The Kullback-Leibler divergence or *relative entropy* is a non-negative measure of dissimilarity between probability distributions.

^3^Hereafter we refer to observations and *hidden states* as *o* and *s*, respectively. We use the more generic term *hidden causes* *x* to refer to *all* aspects of the posterior—including hidden states, policies, and hyperparameters of the generative model.

^4^This threshold is referred to as “residual uncertainty,” and by default is set to as $\frac{1}{64}$ nats.

## Appendix

We provide the derivation of Equation (8), the expected free energy as an upper bound on the negative information gain and negative extrinsic value:

(i) $$\begin{array}{l} G(\tau ,\pi )={𝔼}_{Q(o_{\tau },s_{\tau }|\pi )}[\text{ln }Q(s_{\tau }|\pi )-\text{ln }P(o_{\tau },s_{\tau })]\\ \;={𝔼}_{Q(o_{\tau },s_{\tau }|\pi )}[\text{ln }Q(s_{\tau }|\pi )-\text{ln }P(o_{\tau },s_{\tau })\\ \;+\underset{=0}{\underset{︸}{\text{ln }Q(s_{\tau }|o_{\tau },\pi )-\text{ln }Q(s_{\tau }|o_{\tau },\pi )}}]\\ \;={𝔼}_{Q(o_{\tau },s_{\tau }|\pi )}[\text{ln }Q(s_{\tau }|\pi )-\text{ln }Q(s_{\tau }|o_{\tau },\pi )\\ \;-\text{ln }P(o_{\tau })]+\underset{expected\;KL\;divergence\;\geq 0}{\underset{︸}{{𝔼}_{Q(o_{\tau }|\pi )}[{𝔼}_{Q(s_{\tau }|o_{\tau },\pi )}[\text{ln }\frac{Q(s_{\tau }|o_{\tau },\pi )}{P(s_{\tau }|o_{\tau })}]]}}\\ \;\geq {𝔼}_{Q(o_{\tau },s_{\tau }|\pi )}[\text{ln }Q(s_{\tau }|\pi )-\text{ln }Q(s_{\tau }|o_{\tau },\pi )\\ \;-\text{ln }P(o_{\tau })]\\ \Rightarrow G(\tau ,\pi )\geq -{𝔼}_{Q(o_{\tau }|\pi )}[{\text{D}}_{KL}[Q(s_{\tau }|o_{\tau },\pi )||Q(s_{\tau }|\pi )]]\\ \;-{𝔼}_{Q(o_{\tau }|\pi )}[\text{ln }P(o_{\tau })] \end{array}$$

We also offer a derivation of Equation (9), the formulation of the expected free energy as the sum of “risk” and “ambiguity,” starting from its definition as an upper bound on the (negative) epistemic and instrumental values. We can write **G** for a given future time point τ and policy π as follows:

(ii) $$\begin{array}{l} G(\tau ,\pi )\geq -\underset{Epistemic\;value}{\underset{︸}{{\text{E}}_{Q(o_{\tau }|\pi )}[D_{KL}[Q(s_{\tau }|o_{\tau },\pi )\|Q(s_{\tau }|\pi )]]}}\\ \;-\underset{Instrumental\;value}{\underset{︸}{{\text{E}}_{Q(o_{\tau }|\pi )}[\text{ln }P(o_{\tau })}}]\\ \;=-{𝔼}_{Q(o_{\tau }|\pi )}[D_{KL}[Q(s_{\tau }|o_{\tau },\pi )||Q(s_{\tau }|\pi )]\\ \;+\underset{=0}{\underset{︸}{\text{ln }Q(o_{\tau }|\pi )-\text{ln }Q(o_{\tau }|\pi )}}]-{\text{E}}_{Q(o_{\tau }|\pi )}[\text{ln }P(o_{\tau })]\\ \;=-{𝔼}_{Q(o_{\tau }|\pi )}[{𝔼}_{Q(s_{\tau }|o_{\tau },\pi )}[\text{ln }\frac{Q(s_{\tau }|o_{\tau },\pi )Q(o_{\tau }|\pi )}{Q(s_{\tau }|\pi )Q(o_{\tau }|\pi )}]]\\ \;-{𝔼}_{Q(o_{\tau }|\pi )}[\text{ln }P(o_{\tau })]\\ \;=-{𝔼}_{Q(s_{\tau }|\pi )P(o_{\tau }|s_{\tau })}[\text{ln }\frac{Q(s_{\tau }|\pi )P(o_{\tau }|s_{\tau })}{Q(s_{\tau }|\pi )Q(o_{\tau }|\pi )}]\\ \;-{𝔼}_{Q(o_{\tau }|\pi )}[\text{ln }P(o_{\tau })]\\ \;=\underset{Ambiguity}{\underset{︸}{{𝔼}_{Q(s_{\tau }|\pi )}\left[\text{H}[P(o_{\tau }|s_{\tau })]\right]}}+\underset{Risk}{\underset{︸}{D_{KL}[Q(o_{\tau }|\pi )||P(o_{\tau })]}} \end{array}$$

The above derivation assumes that the mapping from predicted states *Q*(*s*_τ_|π) to predicted observations *Q*(*o*_τ_|*s*_τ_, π) is given as the likelihood of the generative model, i.e., *Q*(*o*_τ_, *s*_τ_|π) = *P*(*o*_τ_|*s*_τ_)*Q*(*s*_τ_|π).

We provide a derivation of Equation (10), the full variational free energy of the posterior over observations, hidden states and policies:

(iii) $$\begin{array}{l} F={\text{E}}_{Q(\tilde{s},\pi )}[\text{ln }Q(\tilde{s},\pi )-\text{ln }P(\tilde{o},\tilde{s},\pi )]\\ \;=-{\text{E}}_{Q(\tilde{s},\pi )}[\text{ln }P(\tilde{o},\tilde{s},\pi )]-\text{H}[Q(\tilde{s},\pi )]\\ \;={\text{E}}_{Q(\pi )}\left[-{\text{E}}_{Q(\tilde{s}|\pi )}[\text{ln }P(\tilde{o},\tilde{s}|\pi )]-\text{H}[Q(\tilde{s}|\pi )]\right]\\ \;+{\text{D}}_{KL}[Q(\pi )||P(\pi )]\\ \;={\text{E}}_{Q(\pi )}[F(\pi )]+{\text{D}}_{KL}[Q(\pi )||P(\pi )] \end{array}$$

## References

[B1] Bastos A. Usrey W. Adams R. Mangun G. Fries P. Friston K. (2012). Canonical microcircuits for predictive coding. Neuron 76, 695–711. 10.1016/j.neuron.2012.10.038 23177956 PMC3777738

[B2] Beal M. J. (2004). Variational algorithms for approximate bayesian inference (Ph.D. thesis), Gatsby Unit, University College London, London, United Kingdom.

[B3] Biehl M. Guckelsberger C. Salge C. Smith S. C. Polani D. (2018). Expanding the active inference landscape: more intrinsic motivations in the perception-action loop. Front. Neurorobot. 12:45. 10.3389/fnbot.2018.00045 30214404 PMC6125413

[B4] Blei D. M. Kucukelbir A. McAuliffe J. D. (2017). Variational inference: a review for statisticians. J. Am. Stat. Assoc. 112, 859–877. 10.1080/01621459.2017.1285773

[B5] Bogacz R. (2017). A tutorial on the free-energy framework for modelling perception and learning. J. Math. Psychol. 76, 198–211. 10.1016/j.jmp.2015.11.003 28298703 PMC5341759

[B6] Bogacz R. Brown E. Moehlis J. Holmes P. Cohen J. D. (2006). The physics of optimal decision making: a formal analysis of models of performance in two-alternative forced-choice tasks. Psychol. Rev. 113, 700–765. 10.1037/0033-295X.113.4.700 17014301

[B7] Felleman D. J. Van D. E. (1991). Distributed hierarchical processing in the primate cerebral cortex. Cereb. Cortex 1, 1–47. 10.1093/cercor/1.1.1 1822724

[B8] Ferro M. Ognibene D. Pezzulo G. Pirrelli V. (2010). Reading as active sensing: a computational model of gaze planning during word recognition. Front. Neurorobot. 4:6. 10.3389/fnbot.2010.00006 20577589 PMC2889689

[B9] Feynman R. (1998). Statistical Mechanics: A Set of Lectures (Advanced Book Classics). Boulder, CO: Westview Press.

[B10] FitzGerald T. H. B. Dolan R. J. Friston K. (2015). Dopamine, reward learning, and active inference. Front. Comput. Neurosci. 9:136. 10.3389/fncom.2015.00136 26581305 PMC4631836

[B11] Friston K. (2008). Hierarchical models in the brain. PLoS Comput. Biol. 4:e1000211. 10.1371/journal.pcbi.1000211 18989391 PMC2570625

[B12] Friston K. (2011). What is optimal about motor control? Neuron 72, 488–498. 10.1016/j.neuron.2011.10.018 22078508

[B13] Friston K. Adams R. A. Perrinet L. Breakspear M. (2012a). Perceptions as hypotheses: saccades as experiments. Front. Psychol. 3:151. 10.3389/fpsyg.2012.00151 22654776 PMC3361132

[B14] Friston K. Buzsáki G. (2016). The functional anatomy of time: what and when in the brain. Trends Cogn. Sci. 20, 500–511. 10.1016/j.tics.2016.05.001 27261057

[B15] Friston K. Daunizeau J. Kiebel S. J. (2009). Reinforcement learning or active inference? PLoS ONE 4:e6421 10.1371/journal.pone.0006421 19641614 PMC2713351

[B16] Friston K. FitzGerald T. Rigoli F. Schwartenbeck P. Pezzulo G. (2017a). Active inference: a process theory. Neural Comput. 29, 1–49. 10.1162/NECO_a_00912 27870614

[B17] Friston K. Kiebel S. (2009). Predictive coding under the free-energy principle. Philos. Trans. R. Soc. B Biol. Sci. 364, 1211–1221. 10.1098/rstb.2008.0300 PMC2666703 19528002

[B18] Friston K. Lin M. Frith C. D. Pezzulo G. Hobson J. A. Ondobaka S. (2017b). Active inference, curiosity and insight. Neural Comput. 29, 2633–2683. 10.1162/neco_a_00999 28777724

[B19] Friston K. Parr T. de Vries B. (2017c). The graphical brain: belief propagation and active inference. Network Neurosci. 1, 381–414. 10.1162/NETN_a_00018 29417960 PMC5798592

[B20] Friston K. Rigoli F. Ognibene D. Mathys C. Fitzgerald T. Pezzulo G. (2015). Active inference and epistemic value. Cogn. Neurosci. 6, 187–214. 10.1080/17588928.2015.1020053 25689102

[B21] Friston K. Rosch R. Parr T. Price C. Bowman H. (2017d). Deep temporal models and active inference. Neurosci. Biobehav. Rev. 77, 388–402. 10.1016/j.neubiorev.2017.04.009 28416414 PMC5461873

[B22] Friston K. Samothrakis S. Montague R. (2012b). Active inference and agency: optimal control without cost functions. Biol. Cybernet. 106, 523–541. 10.1007/s00422-012-0512-8 22864468

[B23] Friston K. Schwartenbeck P. FitzGerald T. Moutoussis M. Behrens T. Dolan R. J. (2013). The anatomy of choice: active inference and agency. Front. Hum. Neurosci. 7:598. 10.3389/fnhum.2013.00598 24093015 PMC3782702

[B24] Girshick A. R. Landy M. S. Simoncelli E. P. (2011). Cardinal rules: visual orientation perception reflects knowledge of environmental statistics. Nat. Neurosci. 14:926 10.1038/nn.2831 21642976 PMC3125404

[B25] Gottlieb J. Oudeyer P.-Y. (2018). Towards a neuroscience of active sampling and curiosity. Nat. Rev. Neurosci. 19, 758–770. 10.1038/s41583-018-0078-0 30397322

[B26] Hassabis D. Maguire E. A. (2007). Deconstructing episodic memory with construction. Trends Cogn. Sci. 11, 299–306. 10.1016/j.tics.2007.05.001 17548229

[B27] Hasson U. Yang E. Vallines I. Heeger D. J. Rubin N. (2008). A hierarchy of temporal receptive windows in human cortex. J. Neurosci. 28, 2539–2550. 10.1523/JNEUROSCI.5487-07.2008 18322098 PMC2556707

[B28] Huang Y. Rao R. P. (2011). Predictive coding. Wiley Interdiscipl. Rev. Cogn. Sci. 2, 580–593. 10.1002/wcs.142 26302308

[B29] Itti L. Baldi P. (2009). Bayesian surprise attracts human attention. Vision Res. 49, 1295–1306. 10.1016/j.visres.2008.09.007 18834898 PMC2782645

[B30] Jóhannesson M. I. Thornton I. M. Smith I. J. Chetverikov A. Kristjánsson r. (2016). Visual foraging with fingers and eye gaze. I-Perception 7:2041669516637279. 10.1177/2041669516637279 27433323 PMC4934673

[B31] Klyubin A. S. Polani D. Nehaniv C. L. (2005). “Empowerment: a universal agent-centric measure of control,” in 2005 IEEE Congress on Evolutionary Computation, Vol. 1 (Edinburgh: IEEE), 128–135. 10.1109/CEC.2005.1554676

[B32] Körding K. P. Wolpert D. M. (2004). Bayesian integration in sensorimotor learning. Nature 427:244. 10.1038/nature02169 14724638

[B33] Lee T. S. Mumford D. (2003). Hierarchical Bayesian inference in the visual cortex. J. Opt. Soc. Am. A 20, 1434–1448. 10.1364/JOSAA.20.001434 12868647

[B34] Linsker R. (1990). Perceptual neural organization: Some approaches based on network models and information theory. Annu. Rev. Neurosci. 13, 257–281. 10.1146/annurev.ne.13.030190.001353 2183677

[B35] March J. G. (1991). Exploration and exploitation in organizational learning. Organ. Sci. 2, 71–87. 10.1287/orsc.2.1.71

[B36] Millidge B. Tschantz A. Seth A. K. Buckley C. L. (2020). On the relationship between active inference and control as inference. arXiv 2006.12964.

[B37] Mirza M. B. Adams R. A. Friston K. Parr T. (2019a). Introducing a bayesian model of selective attention based on active inference. Sci. Rep. 9, 1–22. 10.1038/s41598-019-50138-8 31558746 PMC6763492

[B38] Mirza M. B. Adams R. A. Mathys C. Friston K. (2018). Human visual exploration reduces uncertainty about the sensed world. PLoS ONE 13:e0190429. 10.1371/journal.pone.0190429 29304087 PMC5755757

[B39] Mirza M. B. Adams R. A. Mathys C. D. Friston K. (2016). Scene construction, visual foraging, and active inference. Front. Comput. Neurosci. 10:56. 10.3389/fncom.2016.00056 27378899 PMC4906014

[B40] Mirza M. B. Adams R. A. Parr T. Friston K. (2019b). Impulsivity and active inference. J. Cogn. Neurosci. 31, 202–220. 10.1162/jocn_a_01352 30407133

[B41] Narayanan S. Jurafsky D. (1998). “Bayesian models of human sentence processing,” in Proceedings of the Twelfth Annual Meeting of the Cognitive Science Society (Cambridge, MA), 1–6.

[B42] Ognibene D. Baldassare G. (2014). Ecological active vision: four bioinspired principles to integrate bottom-up and adaptive top-down attention tested with a simple camera-arm robot. IEEE Trans. Auton. Mental Dev. 7, 3–25. 10.1109/TAMD.2014.2341351

[B43] Ognibene D. Demiris Y. (2013). “Towards active event recognition,” in Twenty-Third International Joint Conference on Artificial Intelligence (Beijing).

[B44] Ólafsdóttir I. M. Gestsdóttir S. Kristjánsson A. (2019). Visual foraging and executive functions: a developmental perspective. Acta Psychol. 193, 203–213. 10.1016/j.actpsy.2019.01.005 30660998

[B45] Palmer J. Huk A. C. Shadlen M. N. (2005). The effect of stimulus strength on the speed and accuracy of a perceptual decision. J. Vis. 5:1. 10.1167/5.5.1 16097871

[B46] Parr T. (2020). Inferring what to do (and what not to). Entropy 22:536 10.3390/e22050536 PMC7517030 33286308

[B47] Parr T. Benrimoh D. A. Vincent P. Friston K. (2018). Precision and false perceptual inference. Front. Integr. Neurosci. 12:39. 10.3389/fnint.2018.00039 30294264 PMC6158318

[B48] Parr T. Friston K. (2017). Uncertainty, epistemics and active inference. J. R. Soc. Interface 14:376 10.1098/rsif.2017.0376 PMC5721148 29167370

[B49] Parr T. Friston K. (2018a). The anatomy of inference: generative models and brain structure. Front. Comput. Neurosci. 12:90. 10.3389/fncom.2018.00090 30483088 PMC6243103

[B50] Parr T. Friston K. (2018b). Attention or salience? Curr. Opin. Psychol. 29, 1–5. 10.1016/j.copsyc.2018.10.006 30359960

[B51] Parr T. Friston K. (2018c). The discrete and continuous brain: from decisions to movement-and back again. Neural Comput. 30, 2319–2347. 10.1162/neco_a_01102 29894658 PMC6115199

[B52] Parr T. Friston K. (2019). Generalised free energy and active inference. Biol. Cybernet. 113, 495–513. 10.1007/s00422-019-00805-w 31562544 PMC6848054

[B53] Parr T. Markovic D. Kiebel S. J. Friston K. (2019). Neuronal message passing using mean-field, bethe, and marginal approximations. Sci. Rep. 9:1889. 10.1038/s41598-018-38246-3 30760782 PMC6374414

[B54] Pezzulo G. Rigoli F. Friston K. (2018). Hierarchical active inference: a theory of motivated control. Trends Cogn. Sci. 22, 294–306. 10.1016/j.tics.2018.01.009 29475638 PMC5870049

[B55] Pineau J. Roy N. Thrun S. (2001). “A hierarchical approach to POMDP planning and execution,” in ICML Workshop on Hierarchy and Memory in Reinforcement Learning (Williamstown, MA).

[B56] Puterman M. L. (1995). Markov decision processes: discrete stochastic dynamic programming. J. Oper. Res. Soc. 46, 792–792. 10.2307/2584317

[B57] Quétard B. Quinton J. C. Mermillod M. Barca L. Pezzulo G. Colomb M.. (2016). Differential effects of visual uncertainty and contextual guidance on perceptual decisions: evidence from eye and mouse tracking in visual search. J. Vis. 16, 28–28. 10.1167/16.11.28 27690168

[B58] Ratcliff R. (1978). A theory of memory retrieval. Psychol. Rev. 85:59 10.1037/0033-295X.85.2.59 3406246

[B59] Ratcliff R. McKoon G. (2008). The diffusion decision model: theory and data for two-choice decision tasks. Neural Comput. 20, 873–922. 10.1162/neco.2008.12-06-420 18085991 PMC2474742

[B60] Rayner K. Well A. D. (1996). Effects of contextual constraint on eye movements in reading: a further examination. Psychon. Bull. Rev. 3, 504–509. 10.3758/BF03214555 24213985

[B61] Runyan C. A. Piasini E. Panzeri S. Harvey C. D. (2017). Distinct timescales of population coding across cortex. Nature 548:92. 10.1038/nature23020 28723889 PMC5859334

[B62] Schmidhuber J. (1991). “Curious model-building control systems,” in Proceedings of International Joint Conference on Neural Networks (Singapore), 1458–1463. 10.1109/IJCNN.1991.170605

[B63] Seth A. K. (2015). The Cybernetic Bayesian Brain: From Interoceptive Inference to Sensorimotor Contingencies: From Interoceptive Inference to Sensorimotor Contingencies. Sussex: Open MIND.

[B64] Seth A. K. Tsakiris M. (2018). Being a beast machine: the somatic basis of selfhood. Trends Cogn. Sci. 22, 969–981. 10.1016/j.tics.2018.08.008 30224233

[B65] Shadlen M. N. Newsome W. T. (1996). Motion perception: seeing and deciding. Proc. Natl. Acad. Sci. U.S.A. 93, 628–633. 10.1073/pnas.93.2.628 8570606 PMC40102

[B66] Sridharan M. Wyatt J. Dearden R. (2010). Planning to see: a hierarchical approach to planning visual actions on a robot using POMDPs. Artif. Intell. 174, 704–725. 10.1016/j.artint.2010.04.022

[B67] Stocker A. A. Simoncelli E. P. (2006). Noise characteristics and prior expectations in human visual speed perception. Nat. Neurosci. 9:578 10.1038/nn1669 16547513

[B68] Sutton R. Barto A. (1998). Reinforcement learning: an introduction. Cambridge, MA: MIT Press.

[B69] Tanenhaus M. K. Spivey-Knowlton M. J. Eberhard K. M. Sedivy J. C. (1995). Integration of visual and linguistic information in spoken language comprehension. Science 268, 1632–1634. 10.1126/science.7777863 7777863

[B70] Theocharous G. Murphy K. Kaelbling L. P. (2004). “Representing hierarchical POMDPs as DBNS for multi-scale robot localization,” in IEEE International Conference on Robotics and Automation, 2004. Proceedings. ICRA'04, Vol. 1 (New Orleans, LA: IEEE), 1045–1051. 10.1109/ROBOT.2004.1307288

[B71] Todorov E. (2008). “General duality between optimal control and estimation,” in 2008 47th IEEE Conference on Decision and Control (Cancun), 4286–4292. 10.1109/CDC.2008.4739438

[B72] Trueswell J. C. Tanenhaus M. K. Garnsey S. M. (1994). Semantic influences on parsing: use of thematic role information in syntactic ambiguity resolution. J. Mem. Lang. 33, 285–318. 10.1006/jmla.1994.1014

[B73] Ungerleider L. G. Haxby J. V. (1994). ‘What' and ‘where' in the human brain. Curr. Opin. Neurobiol. 4, 157–165. 10.1016/0959-4388(94)90066-3 8038571

[B74] van den Broek L. Wiegerinck W. Kappen H. J. (2010). “Risk sensitive path integral control,” in 26th Conference on Uncertainty in Artificial Intelligence (UAI 2010) (Catalina Island, CA: AUAI Press).

[B75] Wald A. Wolfowitz J. (1948). Optimum character of the sequential probability ratio test. Ann. Math. Stat. 19, 326–339. 10.1214/aoms/1177730197

[B76] Yang S. C.-H. Lengyel M. Wolpert D. M. (2016). Active sensing in the categorization of visual patterns. eLife 5:e12215. 10.7554/eLife.12215 26880546 PMC4764587

[B77] Yarbus A. L. (1967). Eye Movements and Vision. New York, NY: Plenum Press.

[B78] Zeidman P. Lutti A. Maguire E. A. (2015). Investigating the functions of subregions within anterior hippocampus. Cortex 73, 240–256. 10.1016/j.cortex.2015.09.002 26478961 PMC4686003

[B79] Zeki S. Goodenough O. Zak P. J. (2004). Neuroeconomics. Philos. Trans. R. Soc. Lond. B Biol. Sci. 359, 1737–1748. 10.1098/rstb.2004.1544 15590614 PMC1693452

